# Variation among *S*-locus haplotypes and among stylar RNases in almond

**DOI:** 10.1038/s41598-020-57498-6

**Published:** 2020-01-17

**Authors:** Shashi N. Goonetilleke, Adam E. Croxford, Timothy J. March, Michelle G. Wirthensohn, Maria Hrmova, Diane E. Mather

**Affiliations:** 1 School of Agriculture, Food and Wine, Waite Research Institute, The University of Adelaide, PMB 1, Glen Osmond, SA 5064 Australia; 2 School of Life Sciences, Huaiyin Normal University, Huai’an, 223300 China

**Keywords:** Plant hybridization, Self incompatability

## Abstract

In many plant species, self-incompatibility systems limit self-pollination and mating among relatives. This helps maintain genetic diversity in natural populations but imposes constraints in agriculture and plant breeding. In almond [*Prunus dulcis* (Mill.) D.A. Webb], the specificity of self-incompatibility is mainly determined by stylar ribonuclease (S-RNase) and *S*-haplotype-specific F-box (SFB) proteins, both encoded within a complex locus, *S*. Prior to this research, a nearly complete sequence was available for one *S*-locus haplotype. Here, we report complete sequences for four haplotypes and partial sequences for 11 haplotypes. Haplotypes vary in sequences of genes (particularly *S-RNase* and *SFB*), distances between genes and numbers and positions of long terminal repeat transposons. Haplotype variation outside of the *S-RNase* and *SFB* genes may help maintain functionally important associations between *S-RNase* and *SFB* alleles. Fluorescence-based assays were developed to distinguish among some *S-RNase* alleles. With three-dimensional modelling of five S-RNase proteins, conserved active sites were identified and variation was observed in electrostatic potential and in the numbers, characteristics and positions of secondary structural elements, loop anchoring points and glycosylation sites. A hypervariable region on the protein surface and differences in the number, location and types of glycosylation sites may contribute to determining S-RNase specificity.

## Introduction

Many plant species, including almond [*Prunus dulcis* (Mill.) D.A. Webb] and some other important tree crops, exhibit self-incompatibility (SI); they are unable to set seed from self-pollination or from pollination by genetically identical or genetically similar plants. While biologically important as a means of maintaining population diversity and avoiding inbreeding, self-incompatibility imposes constraints on agricultural and horticultural practices (requiring polliniser varieties) and in plant breeding (restricting the choice of cross combinations). Self-incompatibility can be sporophytic, involving recognition of the genotype of the pollen parent, or gametophytic, involving recognition of the pollen genotype. In sporophytic SI, incompatibility reactions prevent the germination of incompatible pollen grains on the stigma. In gametophytic SI, incompatibility reactions impede the growth of incompatible pollen tubes through the style.

In *Prunus*, including almond, SI is gametophytic and under the genetic control of complex and highly variable *S* loci. Based on the results of experimental crosses, there are thought to be at least 50 variants at the almond *S*-locus^1–5^. Sequencing of a 71,953 bp region of one haplotype (*S*_7_, also known as *S*_*c*_) showed that the almond *S* locus includes *S*-locus F-box (*SLF*), stylar RNase (*S-RNase*) and *S*-haplotype-specific F-box (*SFB*) genes, other open reading frames and pairs of long-terminal-repeat retrotransposons (LTRs)^6^. For this complex locus, variant forms of individual genes are referred to as alleles, while variant forms of the entire locus are referred to as haplotypes. Although the *S*_7_ haplotype is the only one for which a nearly complete sequence has been published, the *SLF*, *S-RNase* and/or *SFB* alleles of some other haplotypes have been fully or partially sequenced^1–5^. Among haplotypes that have been physically mapped, the order and orientations of *S*-locus features are conserved, but the distances between these features vary^6^.

In *Prunus*, including almond, the specificity of SI is mainly determined by the *S-RNase* and *SFB* genes^6,7^, which are expressed in pistils and pollen tubes, respectively. S-RNases act as cytotoxins in self pollen tubes^8^ but the role of SFB proteins is not completely understood. In sweet cherry (*P. avium*), SFB proteins act as ‘blockers’ that protect self S-RNases from detoxification by SFB-like and SLF-like ‘general inhibitor’ proteins^9,10^. This allows self S-RNases to remain active and capable of arresting pollen tube growth. It is not known which, if any, F-box proteins play general inhibitor roles in the SI system of almond.

A few almond cultivars are self-fertile. This phenotype has been attributed to a dominant *S-RNase* allele, designated *S*_*f*_^11,12^ or *S*_*fi*_ (*S*_*f*_-*inactive*)^13^. Plants carrying this allele do not express an active S_f_-RNase, possibly due to poor transcription^14^, and are not able to block the growth of *S*_*f*_ pollen tubes. A similar allele, *S*_*fa*_ (*S*_*f*_*-active*) expresses an active S-RNase and confers SI^13^. Despite their contrasting phenotypes, *S*_*fi*_- and *S*_*fa*_*-RNase* alleles have identical nucleotide sequences and are linked with identical *SFB* alleles^14^. This apparent paradox was resolved by the discovery that *S*_*fi*_ and *S*_*fa*_ are epialleles^15^, differing by the methylation of a single nucleotide upstream of the coding sequence. This epigenetic difference may determine whether the *S*_*f*_*-RNase* allele is expressed. Consistent with this interpretation, *S*_*fi*_*S*_*fa*_ heterozygotes have been found to be fully self-incompatible^16^, with their *S*_*fa*_-RNase able to block the growth of both *S*_*fi*_ and *S*_*fa*_ pollen tubes.

To further investigate variation among *S*-locus haplotypes, we amplified and sequenced PCR products from 48 diverse almond clones. To improve the efficacy of *S* allele detection, we developed simple fluorescence-based marker assays to distinguish among *S-RNase* alleles. To investigate how structural features might affect S-RNase function and specificity, we conducted three-dimensional (3D) protein modelling for the predicted products of five *S-RNase* alleles and investigated how sequence variation in a highly variable region could affect domain structure, glycosylation and physical interacting forces that might influence the specificity of SI.

## Results

### *S*-locus haplotype sequences

From Illumina paired-end sequence data generated for a pooled library of products amplified from the *S* locus of diverse almond clones (Supplementary Table S1), sequences from each of eight clones known to carry the *S*_7_ haplotype were extracted and aligned with the previously available *S*_7_ haplotype sequence (AB081587). An *S*_7_ haplotype sequence was assembled for each clone (Supplementary Table S2). Among these haplotypes, there were sequence discrepancies at just eight of 71,953 nucleotide positions. Haplotype sequences from the cultivars Keanes (MH029539) and Capella (MH029540) were complete and were identical to each other. Their sequence was selected as the consensus sequence for *S*_7_. It includes the identity of 21 nucleotides that were missing from AB081587. It differs from AB081587 at just five positions. At all five of those positions, the same nucleotide was called for all eight clones.

Using the consensus *S*_7_ sequence as a new reference for *S*_7_, haplotype sequences were obtained for: *S*_1_, from Brown Nonpareil (*S*_1_*S*_7_); *S*_8_, from Nonpareil and McKinlays (both *S*_7_*S*_8_); and *S*_*f*_, from the self-fertile clones Carina, Mira, Capella, T5 and T7 (all *S*_7_*S*_*f*_). There is just 2% inconsistency between the two *S*_8_ sequences and 3% inconsistency among the five *S*_*f*_ sequences. Among the four fully-sequenced haplotypes (*S*_1_, *S*_7_, *S*_8_ and *S*_*f*_), positional sequence identity ranges from 51 to 84% (Supplementary Table S3).

**Figure 1:**
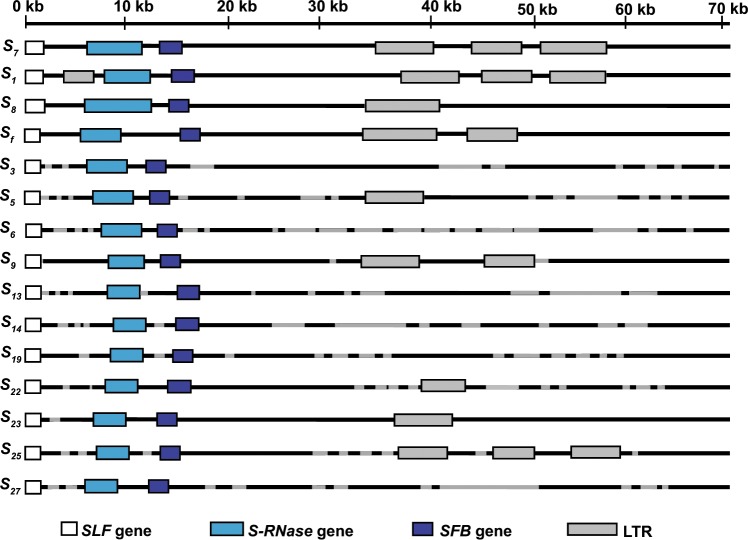
*S*-locus structure. Structure of the almond *S* locus showing the positions of the *SLF*, *S-RNase* and *SFB* genes and long terminal repeat retrotransposons (LTRs). Black lines indicate regions for which sequences were obtained and grey lines indicate gaps in the sequence.

Using the four complete haplotype sequences as references, partial sequences were derived for other haplotypes (*S*_3_, *S*_5_, *S*_6_, *S*_9_, *S*_13_, *S*_14_, *S*_19_, *S*_22_, *S*_23_, *S*_25_ and *S*_27_). The total lengths of the sequences obtained for these haplotypes range from 47% (*S*_6_) to 99% (*S*_23_) of the length of the *S*_7_ haplotype sequence (Fig. 1).

In each of the completely sequenced haplotypes, 12 or more open reading frames (ORFs) were detected: 12 in *S*_7_, 12 in *S*_8_, 14 in *S*_1_ and 18 in *S*_*f*_. (Supplementary Table S4; Supplementary Fig. S1). In each case, these include ORFs that correspond with the *SLF*, *S-RNase* and *SFB* genes. For some ORFs (including ten in the *S*_*f*_ haplotype and the last ORF in each haplotype), no similarity to known-function genes was detected. For others (five for *S*_7_ and *S*_*f*_, six for *S*_1_ and eight for S_8_), homology with transposases from other *Prunus* species was detected. Most of these transposases belong to the *Ty*1*-copia* RNase family and contain a DDE motif.

For the interval between the *S-RNase* and *SFB* genes, complete sequences were obtained for 11 haplotypes (*S*_*1*_, *S*_3_, *S*_5_, *S*_7_, *S*_8_, *S*_9_, *S*_22_, *S*_23_, *S*_25_, *S*_27_ and *S*_*f*_). These sequences, which are AT-rich (65–70%), range in length from 1.2 (*S*_9_) to 6.6 kb (*S*_*f*_). The sequence identity among them ranges from 21% (between *S*_1_ and *S*_*f*_) to 98% (between *S*_7_ and *S*_23_) (Supplementary Table S5).

Pairs of LTRs were detected within each haplotype, mostly in positions that correspond to the LTR-containing region of the *S*_7_ haplotype (Fig. 1). No LTRs were detected within the *SLF*, *S-RNase* or *SFB* genes or between the *S-RNase* and *SFB* genes, but in the *S*_1_ haplotype, an LTR pair was detected between the *SLF* and *S-RNase* genes. All of the LTRs detected here are *Ty*1*-copia*-like retrotransposons with TG/CA boxes in their 5ʹ and 3ʹ ends. Their protein-binding sites are TyrGTA, IleAAT, MetCAT and AlaTGC.

### *SLF*, *S-RNase* and *SFB* allele sequences

The *SLF* gene, which is about 1.2 kb long and has no introns, was sequenced for all 15 haplotypes. Pairwise sequence identities among *SLF* alleles are high, ranging from 70 to 98% (Supplementary Table S6). Among the predicted products of the 15 *SLF* alleles, 210 of 325 amino acid residues are absolutely conserved across all 15 alleles (Supplementary Fig. S2). Sequence comparisons with SLF-like proteins from sweet cherry^10^ showed that the predicted products of all 15 almond *SLF* alleles products are most similar to PavSLFL1 (sequence identity between 82 and 95%, compared to between 55 and 61% for PavSLFL4/5 and no more than 36% for any other PavSLFL) (Supplementary Table S7). Comparisons of PavSLFL sequences with a pseudomolecule sequence for almond chromosome 6 revealed possible homologs of PavSLFLs near the almond *S*-locus: Prudul26A008798 (97% sequence identity with PavSLFL2), Prudul26A009208 (95% sequence identity with PavSLFL3), Prudul26A016695 (95% sequence identity with PavSLFL6) and Prudul26A015917 (97% identity with PavSLFL8).

**Figure 2:**
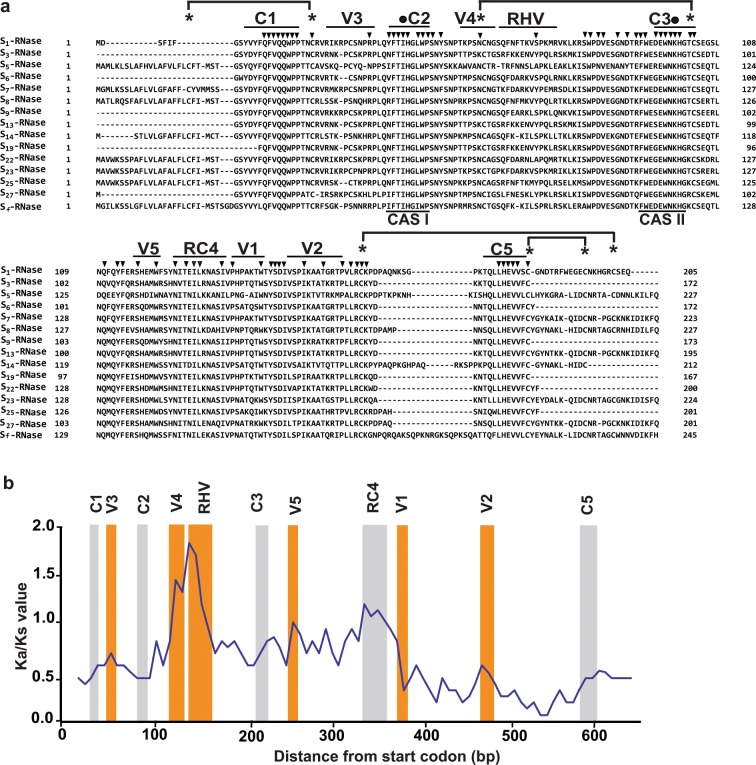
S-RNase sequence alignment and Ka/Ks ratios. (**a**) Sequence alignment of 15 almond S-RNases, showing conserved regions (C1, C2, C3, RC4 and C5), variable regions (V1–V5) a hypervariable region (RHV) and conserved active segments (CAS I and CAS II). Positions of absolutely conserved residues, conserved histidine residues and conserved cysteine residues are indicated by arrowheads, circles and asterisks, respectively. Cysteine residues that are predicted to be linked by disulphide bridges are connected by lines. (**b**) Mean nonsynonymous/synonymous (Ka/Ks) ratios for coding regions of the *S-RNase* gene of almond, average Ka/Ks values calculated for 100 bp sliding windows with a 20 bp step size. Conserved regions (C1, C2, C3, RC4 and C5) are highlighted in grey and variable (V1–V5) or hypervariable (RHV) regions in the S-RNase are highlighted in orange.

The *S-RNase* gene, which was completely sequenced for 11 haplotypes (*S*_*1*_, *S*_5_, *S*_7_, *S*_8_, *S*_13_, *S*_14_, *S*_23_, *S*_25_, *S*_27_ and *S*_*f*_) and partially sequenced for four haplotypes (*S*_3_, *S*_6_, *S*_9_ and *S*_19_), is much more variable. The completely sequenced *S-RNase* alleles range in length from 1.0 kb (*S*_1_) to 4.5 kb (*S*_8_). Their pairwise nucleotide sequence identities range from 19% (between *S*_1_ and *S*_8_) to 51% (between *S*_7_ and *S*_27_) (Supplementary Table S8). Differences among alleles include both sequence differences within exons and length polymorphisms within introns (especially intron 2). The deduced protein sequences of the completely sequenced *S-RNase* alleles contain previously reported conserved regions (C1, C2, C3, RC4 and C5) and variable regions (RHV, V1 and V2) (Fig. 2a). Three additional variable regions were identified: V3 between C1 and C2; V4 between C2 and RHV; and V5 between C3 and RC4 (Fig. 2a). The nonsynonymous-to-synonymous ratio (Ka/Ks) for these alleles is 0.60 (Ka = 0.15, Ks = 0.25), with most codon differences occurring within V1, V2 and RHV (Fig. 2b).

The *SFB* gene was completely sequenced for 11 haplotypes (*S*_1_, *S*_3_, *S*_5_, *S*_7_, *S*_8_, *S*_9_, *S*_22_, *S*_23_, *S*_25_, *S*_27_ and *S*_*f*_) and partially sequenced for four haplotypes *(SFB*_6_, *SFB*_13_, *SFB*_14_ and *SFB*_19_)_._ The completely sequenced alleles range in length from 1.1 kb (*S*_9_) to 1.5 kb (*S*_1_). Their pairwise sequence identities range from 35% (between *SFB*_7_ and *SFB*_*f*_) to 86% (between *SFB*_1_ and *SFB*_23_) (Supplementary Table S9). The *SFB* gene has one intron, which is within its 5ʹ untranslated region and is less polymorphic than either of the *S-RNase* introns. In protein sequences deduced from complete *SFB* allele sequences, several previously reported features of the protein are evident: an F-box motif, two variable regions (V1 and V2) and two hypervariable regions (HVa and HVb) (Supplementary Fig. S3). Two additional short highly variable regions (V3 and V4) were detected, both between V1 and V2. Within the F-box motif, the *SFB*_6_*, SFB*_13_*, SFB*_14_ and *SFB*_19_ proteins each have an insertion of a single arginine, while *SFB*_23_ and *SFB*_27_ each have a deletion of eight amino acid residues. Within V1, many amino acid residues are conserved among the alleles examined here. An overall Ka/Ks ratio of 0.50 (Ka = 0.11, Ks = 0.22) was computed using the complete *SFB* gene sequences. Most of the non-synonymous changes are in the hypervariable regions HVa and HVb, within which Ka/Ks values range from 0.9 to 1.5 (Supplementary Fig. S3), but there is also considerable variation in Ka/Ks values in the F-box motif, ranging from 0.5 to 0.9.

### Marker assays to distinguish among *S-RNase* alleles

**Figure 3:**
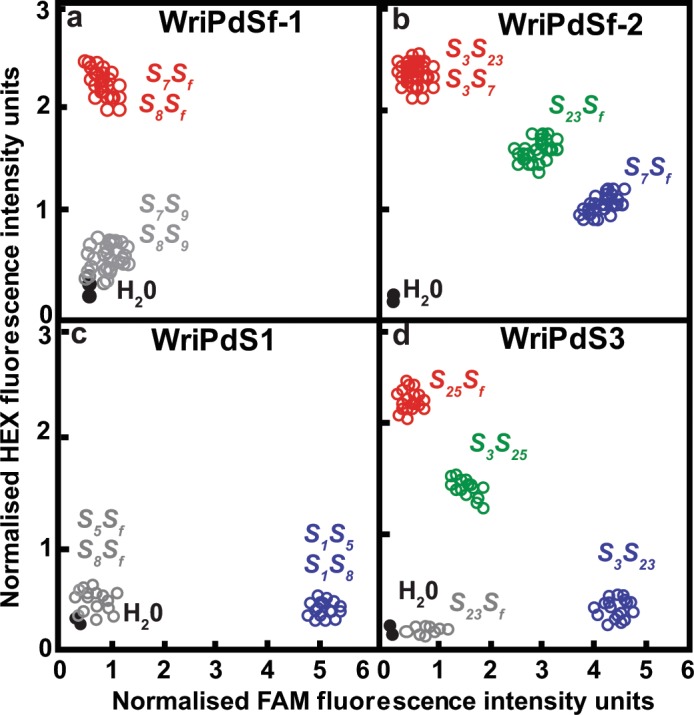
Marker assay results. Results obtained for (**a**) Nonpareil (*S*_7_*S*_8_) × Vairo (*S*_9_*S*_*f*_) F_1_ progeny using primer set WriPdSf-1, (**b**) Chellaston (*S*_7_*S*_23_) × Lauranne (*S*_3_*S*_*f*_) F_1_ progeny using primer set WriPdSf-2, (**c**) Carmel (*S*_5_*S*_8_) × 12–350 (*S*_1_*S*_*f*_) F_1_ progeny using primer set WriPdS1 and (**d**) Johnston’s Prolific (*S*_23_*S*_25_) × Lauranne (*S*_3_*S*_*f*_) F_1_ progeny using primer set WriPdS3. Data shown are intensities of FAM and HEX fluorescence, each normalised against fluorescence from an internal ROX reference.

To provide a presence-absence assay for the *S*_*f*_*-RNase* allele, a primer pair (WriPdSf-1; Supplementary Table S10) was designed for an *S*_*f*_-specific site within intron 2 of the *S-RNase* gene (Supplementary Fig. S4). With this assay, fluorescence is detected for the *S*_*f*_ allele and no signal is detected for any of the other alleles. For example, when this assay was applied to Nonpareil (*S*_7_*S*_8_) × Vairo (*S*_9_*S*_*f*_) F_1_ progeny, HEX fluorescence was detected for *S*_7_*S*_*f*_ and *S*_8_*S*_*f*_ progeny and little or no fluorescence was detected for *S*_7_*S*_9_ or *S*_8_*S*_9_ progeny (Fig. 3a).

Four additional primer sets (Supplementary Table S10) were designed to query an A/C SNP that distinguishes *S*_*f*_ (A) from each of the SI alleles considered here (all C) (Supplementary Fig. S5). Each of these sets includes a primer in the conserved region C1 and two allele-specific primers that overlap with part of the conserved region C2.

In primer set WriPdSf-2, the FAM-tailed primer is exactly complementary to the *S*_*f*_ sequence throughout the annealing site. The HEX-tailed primer is exactly complementary to the *S*_3_, *S*_9_, *S*_23_ and *S*_25_ sequences throughout the annealing site but not to the *S*_1_*, S*_5_*, S*_7_ and *S*_8_ sequences. For *S*_5_, the first mismatch is too far from the target SNP to interfere with annealing and amplification. For *S*_1_, *S*_7_ and *S*_8_, the mismatches are close enough to the target SNP to prevent annealing and amplification. When the WriPdSf-2 primer set was applied to Chellaston (*S*_7_*S*_23_) × Lauranne (*S*_3_*S*_*f*_) F_1_ progeny, HEX fluorescence was detected from the HEX-HEX genotype *S*_3_*S*_23_ (half from each allele) and from the null-HEX genotype *S*_3_*S*_7_ (all from *S*_3_), FAM fluorescence was detected for the null-FAM genotype *S*_7_*S*_*f*_ (all from *S*_*f*_) and both HEX and FAM fluorescence were detected for the HEX-FAM genotype *S*_23_*S*_*f*_ (HEX from *S*_23_ and FAM from *S*_*f*_) (Fig. 3b). As is normally expected for KASP markers, the total amount of HEX fluorescence for HEX-HEX genotypes was about the same as for null-HEX genotypes and about twice that for HEX-FAM genotypes. In summary, this primer set successfully discriminated *S*_*f*_ genotypes from all tested non-*S*_*f*_ genotypes, while also discriminating among some *S*_*f*_ genotypes and among some non-*S*_*f*_ genotypes.

The primer sets WriPdSf-3, WriPdSf-4 and WriPdSf-5 are similar to WriPdSf-2 but include degenerate allele specific primers, to accommodate polymorphisms other than the target SNP. For each of these primer sets, *S*_*f*_ is the only allele for which FAM fluorescence is detected. In each case, there are some SI alleles for which HEX fluorescence is detected and at least one SI allele for which little or no fluorescence is detected. Application of WriPdSf-2, WriPdSf-3, WriPdSf-4 and WriPdSf-5 to synthesised DNA representing the alleles *S*_*f*_, *S*_1_, *S*_3_, *S*_5_, *S*_7_, *S*_8_, *S*_9_, *S*_23_ and *S*_25_ and to mixtures of synthetic DNA representing heterozygous combinations of these alleles confirmed that all four primer sets yield FAM fluorescence when *S*_*f*_ is present and HEX fluorescence when any of *S*_3_, *S*_5_, *S*_9_, *S*_23_ or *S*_25_ are present (Supplementary Fig. S6). In each case the amount of HEX fluorescence obtained for one allele with a mismatch (*S*_5_) was very similar to that detected for the alleles with no mismatches (*S*_3_, *S*_9_, *S*_23_ and *S*_25_). In addition, WriPdSf-3 yields HEX fluorescence when *S*_1_ or *S*_8_ is present and WriPdSf-4 yields HEX fluorescence when *S*_7_ is present. When these markers were tested on synthetic DNA representing alleles for which neither HEX nor FAM fluorescence was expected, there was some HEX fluorescence detected (Supplementary Fig. F6), indicating that with an abundance of template DNA, some annealing of the HEX-tailed primer occurred despite mismatches in the annealing site. Nevertheless, these data points were well separated from those for which HEX fluorescence was expected. When these markers were tested on mapping populations segregating for null alleles, the results were exactly as expected (Supplementary Fig. S7): HEX fluorescence for null-HEX heterozygotes (*S*_1_*S*_7_ and *S*_7_*S*_23_ for WriPdSf-3; *S*_1_*S*_5_ for WriPdSf-4). FAM fluorescence for null-FAM heterozygotes (*S*_7_*S*_*f*_ for WriPdSf-2 and WriPdSf-5; *S*_8_*S*_*f*_ for WriPdSf-2, WriPdSf-4 and WriPdSf-5), both HEX and FAM fluorescence for HEX-FAM heterozygotes (*S*_1_*S*_*f*_ and *S*_23_*S*_*f*_ for WriPdSf-3 and *S*_5_*S*_*f*_ for WriPdSf-4) and a low levels of HEX fluorescence for null-null heterozygotes (*S*_1_*S*_7_ for WriPdSf-2 and WriPdSf-6; *S*_1_*S*_8_ for WriPdSf-2, WrPdSf-4 and WriPdSf-5).

To provide assays to distinguish among *S-RNase* alleles that confer SI, ten additional primer sets were designed. Seven of these (WriPdS1, WriPdS5, WriPdS7-2, WriPdS8, WriPdS9, WriPdS23 and WriPdS25-2) consist of just two primers each (Supplementary Fig. S8). They provide presence-absence assays with which FAM fluorescence is detected for the target allele (e.g. *S*_1_ in Fig. 3c) and little or no fluorescence is detected when the target allele is not present. The other three assays (WriPdS3, WriPdS7-1 and WriPdS25-1) consist of three primers each (Supplementary Fig. S9) and can distinguish FAM target alleles from HEX target allele(s) (e.g. *S*_3_ vs *S*_25_ in Fig. 3d).

Results from application of the 15 primer sets to a variety panel with known *S* genotypes and to the progeny of appropriate crosses are shown in Supplementary Fig. S10. In each set of results, there was significant variation (*p* < 0.001) among genotypic clusters defined based on FAM and HEX fluorescence intensities (Supplementary Tables S11 and S12). For members of the variety panel, there were no inconsistencies between prior genotypic information and the clusters to which they were assigned. Across a total of 3,417 progeny that were assigned to genotypic classes and for which observed genotypic ratios were compared to expected ratios using a chi-square test (χ^2^, α = 0.05), there were no statistically significant deviations from the expectation that half of the progeny would carry the target *S* allele (Supplementary Table S13). In populations that were analysed with more than one marker, there were no inconsistencies in results among markers. On trees that had been genotyped as self-fertile and for which branches were bagged to exclude foreign pollen, fruits were consistently set on the bagged branches (Supplementary Table S14).

### 3D models of S-RNase proteins

**Figure 4:**
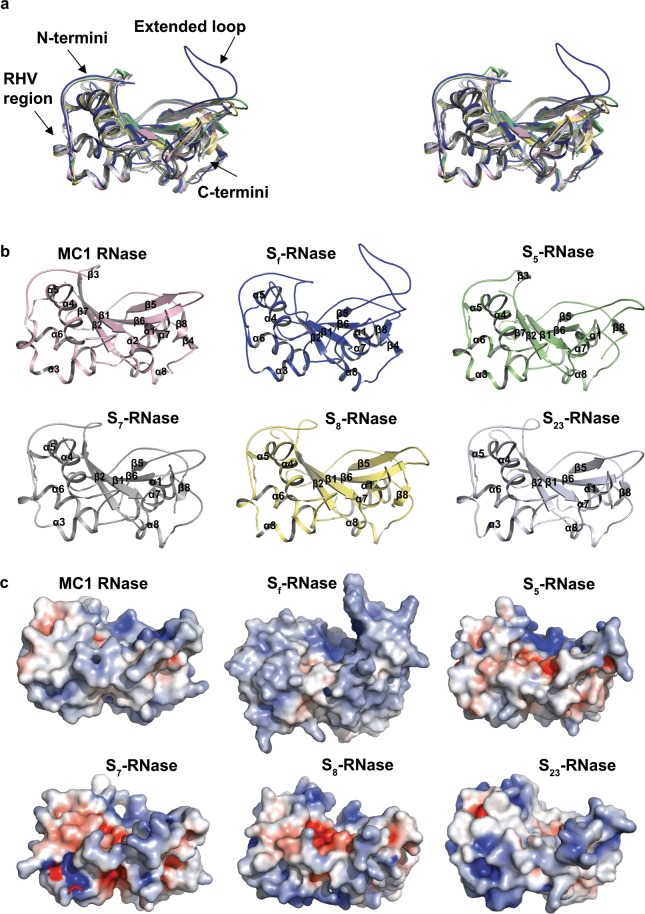
Molecular properties of almond S-RNase structural models. (**a**) Stereo representation of the superposition of 3D structures of S-RNases, whereby the template crystal structure (the MC1 RNase from seeds of bitter gourd) is in pink and the 3D models of almond S_f_-RNase, S_5_-RNase, S_7_-RNase, S_8_-RNase and S_23_-RNases are blue, green, grey, yellow and tint blue, respectively. Almond structural models were superposed on the template structure with RMSD values in the range of 0.15 Å to 0.19 Å for 179 C^α^ atoms. (**b**) Dispositions of secondary structure elements in the template, with the S_f_-, S_5_-, S_7_-, S_8_- and S_23_-RNases indicated in pink, blue, green, grey, yellow and tint blue, respectively. (**c**) Molecular surface morphologies of the template structure, and almond S_f_-, S_5_-, S_7_- S_8_- and S_23_-RNase models coloured by electrostatic potentials display electroneutral (white), electropositive (blue, contoured at +5 kilotesla einstein^−1^) and electronegative (red, contoured at −5 kilotesla einstein^−1^) regions, and presented in the same orientations as the cartoons in panel.

PSI-BLAST searches yielded eight candidate templates (1J1G, 1J1F, 1BK7, 1UCA, 1UCC, 1UCD, IUCG and 1V9H) for almond S-RNases, all with protein sequence identities above 35% and similarities of 47% or 48%. Among these candidate templates, 1J1G (for the MC1 RNase isolated from seeds of bitter gourd (*Momordica charantia* L.) was selected for generating 3D models for the S_5_-, S_7_-, S_8_-, S_23_- and S_f_-RNases. The MC1 protein sequence has 38% identity and 48% similarity to the S_7_-RNase and S_23_-RNase protein sequences (E-value = 1.71e^−27^) and 36% identity and 47% similarity to the S_f_-RNase (E-value = 1.72e^−27^), S_5_-RNase (E-value = 1.69e^−27^) and S_8_-RNase (E-value = 1.71e^−27^) protein sequences. The first 22 residues at the N-termini of the almond S-RNases could not be modelled because no suitable structural template was identified for this region. Modelling of the remaining protein sequences indicated that the folding topologies of the almond S-RNases are similar to those of the template protein and other T2 RNases^17^. All of these RNases consist of α-helices and β-strands that are inter-connected by loops (Fig. 4) and can be classified in family d.124.1.1 of the SCOPe 2.06 database^18^.

Among models generated based on alternative alignments, no differences were found in the locations of α-helices and β-sheets, DOPE values or MOF values. Based on Ramachandran plots, none of the residues of any 3D models are positioned in disallowed regions. All models can therefore be considered to have satisfactory stereo-chemical quality. The G factor values of the models range from 0.25 (S_23_-RNase) to 0.33 (S_8_-RNase), compared to 0.35 for the template structure (Supplementary Table S15). Analysis with ProSa 2003 indicated that the conformational energies of residues are in negative regions in all models. For these reasons, all structural models can be deemed to be correct.

The 1J1G template structure has eight α-helices and eight β-sheets, while each of the protein structures generated for almond S-RNases has seven α-helices and between five and seven β-sheets (five for S_7_-, S_8_-, and S_23_-RNases, six for S_f_-RNase and seven for S_5_-RNase) (Fig. 4a,b; Supplementary Fig. S11). In all five S-RNases, the α-helices are located in approximately the same positions. They range in length from six to 15 residues, with α_5_ being the longest in all cases. Within the S_5_-, S_7_-, S_8_- and S_23_-RNases, four β-sheets (β_1_, β_2_, β_5_ and β_6_) are located in approximately the same positions. Three of these (β_1,_ β_2_, and β_6_) form an antiparallel β-sheet that packs well with α-helices in the interior of the molecule. The overall molecular dimensions of each S-RNase are approximately 50 Å × 40 Å × 30 Å. The estimated solvent-accessible surface areas of the predicted almond S-RNases are 78% for the S_5_-, S_7_-, S_8_- and S_23_-RNases and 81% for the S_f_-RNase. The percentages of the exposed surface occupied by positively charged residues are 22%, 25%, 25%, 23% and 19% for the S_5_-, S_7_-, S_8_-, S_23_- and S_f_-RNases, respectively. Electropositive regions (RHV, V1, V2 and V4) of the S-RNases have high Ka/Ks ratios (ranging from 1.2 to 1.8). Those regions have higher average exposed surface (25%) than neutral and negatively charged regions (10%).

Each of the five S-RNase proteins modelled here has eight conserved cysteine residues. These residues are predicted to form four disulphide bridges (Fig. 2). These connect a region upstream of C1 with a region between C1 and V3; V4 with C3, C5 with a region downstream of V2 and two regions downstream of C5 with each other.

**Figure 5:**
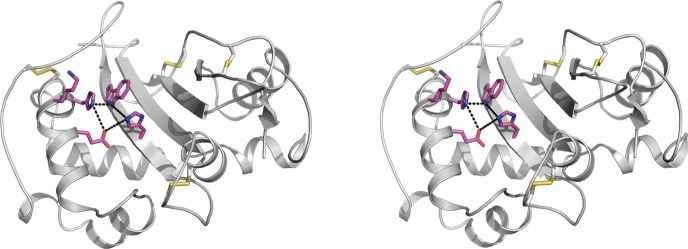
Stereo representation of the active site residues of the almond S_7_-RNase. Active site residues (His40, Trp43 in C2 region and Glu93, Lys96, His97 in C3 region) are shown in cpk magenta on the background of the cartoon model (in grey). The positions of disulphide bridges (Cys20-Cys28, Cys56-Cys100, Cys161-Cys192, Cys176-Cys187) are shown in yellow. Distances among the active site residues are shown with dashed lines.

Comparisons with the S_3_-RNase of Japanese pear enabled identification of putative active sites within almond S-RNases^19^. These sites include conserved cysteine, histidine, glutamic acid, lysine and tryptophan residues, which are separated by distances ranging from 3.2 Å to 5.0 Å (dashed lines in Fig. 5).

In all five S-RNases, the variable regions V3, V4 and V5 and the hypervariable region RHV are exposed on the protein surface. In the S_7_-, S_8_-, and S_23_-RNases, V4 is highly positively charged (Fig. 4c). Considerable structural variation was detected in RHV, which consists of 18 residues in the S_5_-, S_7_-, S_8_- and S_23_-RNases but only 14 residues in the S_f_-RNase. In the S_7_-, S_8_-, and S_23_-RNases, RHV consists of two α-helices (α_3_ and α_4_) and a short loop, while in the S_f_- and S_5_-RNases, it has only one α-helix (α_4_) and a short loop. The residues in the RHV loop-anchoring points vary in size, charge, polarity and hydrophobicity. Notably, the last residue of each RHV loop-anchoring point is polar: glutamine in the S_5_-RNase, asparagine in the S_7_-RNase, tyrosine in the S_8_-RNase and serine in the S_23_- and S_f_-RNases (Supplementary Table S16).

Among the five S-RNase proteins that were modelled here, the most obvious structural difference involves a loop located between the variable region V2 and the conserved region C5. In the S_f_-RNase, this loop is much longer (30 residues) than in other S-RNase proteins (10 to 20 residues) (Fig. 4), with eight S_f_-specific residues: an isoleucine, an asparagine, a glycine, a phenylalanine, an alanine and three glutamines. The average exposed surface of the extended loop region of the S_f_-RNase is 16%.

**Figure 6:**
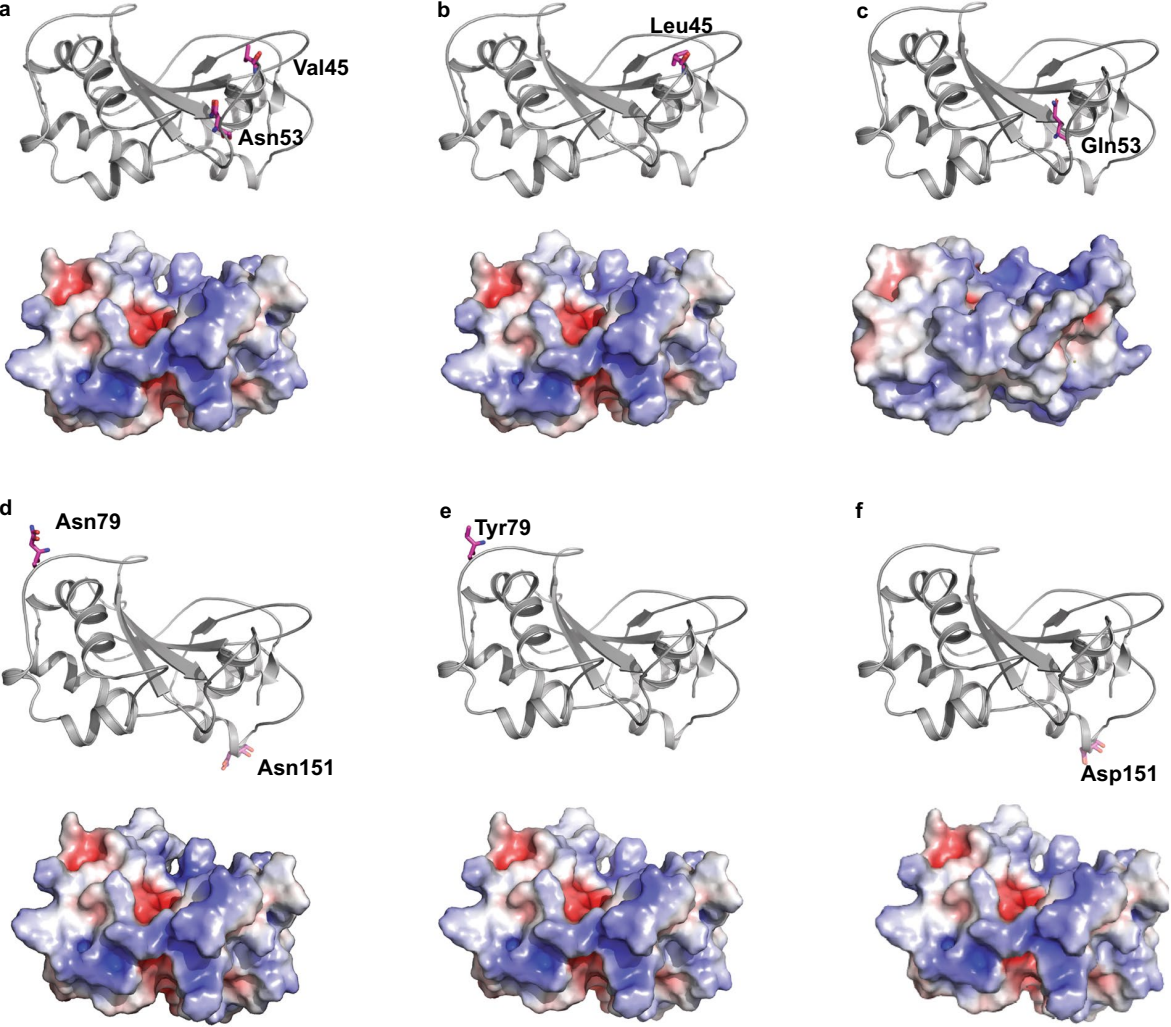
Examples of effects of the mutation of individual S_7_-RNase residues. (**a**) Wild type S_7_-RNase showing the positions of Val45 and Asn53 residues. (**b**) Mutant S_7_-RNase with Val45 replaced by Leu45, resulting in an energy loss of 1.58 kcal/mol. (**c**) Mutant S_7_-RNase with Asn53 replaced by Gln53, resulting in an energy gain of 5.1 kcal/mol. (**d**) Wild-type S_7_-RNAse showing the positions of Asn79 and Asn151 residues. (**e**) Mutant S_7_-RNase with Asn79 replaced by Tyr79, resulting in an energy loss of 0.18 kcal/mol (**f**) Mutant S_7_-RNase with Asn151 replaced by Asp151, resulting in an energy gain of 0.12 kcal/mol. The lower part of each panel presents protein surfaces coloured by electrostatic potentials: blue = + 5 kT·e^−1^; white = neutral; red = −5 kT·e^−1^. Structures are shown in the same orientations as in panels a and b of Fig. 4.

With *in silico* mutation of individual amino acid residues of the S_7_-RNase by the corresponding residues present at the same positions in other almond S-RNases (Supplementary Fig. S12), it was possible to estimate relative changes in unfolding enthalpy values (∆∆G) due to specific mutations (Fig. 6; Supplementary Fig. S12). Most of the mutations were destabilising but a few were classified as highly stabilising (∆∆G < −1.84 kcal/mol). Most of the highly destabilising mutations (∆∆G >1.84 kcal/mol) were at positions near the N- or C-termini.

Between four and seven *N*-glycosylation sites were identified in each of the S_5_-, S_7_-, S_8_-, S_23_- and S_f_-RNases (Supplementary Fig. S11). Many of these sites were within loops, and none were within β-sheets. Two types of sequon were detected: Asn-Xaa-Thr and Asn-Xaa-Ser, where Xaa is any amino acid residue except proline. Only one of these (an Asn-Ile-Thr sequon in the RC4 region) is conserved among all five S-RNases. C-terminal regions had higher Asn-Xaa-Thr to Asn-Xaa-Ser ratios than N-terminal regions. Substitution of Asn residues within S_7_-RNase sequons did not substantially affect electrostatic potential (Fig. 6), but substitution of Asn residues within some S_7_-RNase sequons affected ∆∆G values. For example, replacement of Asn79 by Try79 caused an energy loss of 0.18 kcal/mol and replacement of Asn151 by Asp151 caused an energy gain of 0.12 kcal/mol. In each of the S_5_-, S_7_- and S_8_-RNases, one *O*-glycosylation site was detected in the V2 region (Supplementary Fig. S12). No *O*-glycosylation sites were detected in either the S_f_- or S_23_-RNases.

## Discussion

Given the complexity of the *S* locus, the extent of variation among haplotypes, the heterozygosity of the clones used here and the availability of just one reference sequence, it was difficult to obtain uniform DNA amplification across the entire *S* locus from all haplotypes and samples. Given that a high level of heterozygosity was expected, some primers were designed with degenerated 3ʹ-end sequences. These primers tended to have low PCR sensitivity and a high degree of non-specific binding, and most of them were not selected for use to obtain amplicons for sequencing. Although SI enforces *S*-locus heterozygosity, some primer pairs seemed to yield only one product from some clones. This could be due to lack of length polymorphism between alleles (generating two products of equal length), sequence polymorphism at primer annealing sites (generating just one product) and/or preferential amplification of some products (generating predominantly one product). It was particularly difficult to obtain useful amplicons for the region in which the previously sequenced haplotype was known to contain LTRs.

The complete consensus *S*_7_ sequence obtained here based on data from eight clones provided minor improvements over the AB081587 sequence and provided new reference upon which an iterative process could be undertaken to assemble complete or partial sequences for other haplotypes. With these sequences, we were able to investigate sequence diversity throughout the locus.

Consistent with difficulties that were experienced in obtaining amplicons, the LTR-containing region was the least completely sequenced. In our *S*_7_ consensus sequence, one of four previously reported LTRs (LTR0) was not detected. This may have been due to the use of different reference sequences (Arabidopsis here, but rice in the previous work^6^). In other haplotypes, between one and four LTR pairs were detected, almost all in approximately the same region of the locus in which LTRs were detected in the *S*_7_ haplotype. No LTRs were detected within the *S-RNase* or *SFB* genes. This is in contrast to Japanese apricot (*Prunus mume* Siebold & Zucc.), for which LTR insertions in the *SFB* gene have been reported to lead to breakdown of SI^20^. While there is no evidence that *S*-locus LTRs are functionally relevant, they may be genetically relevant, with variation in their numbers, lengths and positions contributing to maintaining tight associations between *S-RNase* and *SFB* alleles.

All 12 ORFs that had previously been reported in the *S*_7_ haplotype^6^ were detected in our *S*_7_ consensus sequence, and up to 18 ORFs were detected in other haplotypes. These included the ORFS for the *SLF*, *S-RNase* and *SFB* genes and ORFs with high homology with known DDE RNase transposes from other *Prunus* species. The catalytic domains of DDE transposases are known to exhibit considerable sequence variability^21,22^, possibly reflecting different ways of recognising transposon DNA and leading to non-specific and/or weak DNA binding activity. The variable number of ORFs among *S*-locus haplotypes and differences in transposon DNA recognition and/or DNA binding ability may contribute to maintaining the specificity of SI in almond. In other *Prunus* species, insertion of transposable elements into *S-RNase* and *SFB* genes has been reported to lead to breakdown of SI^20,23,24^, but no such insertions were observed here.

This work increased the number of sequenced *SLF* alleles from just two (*SLF*_7_ and *SLF*_8_) to fifteen. Consistent with the expectation that SLF does not affect SI specificity in *Prunus* spp.^6^, pairwise sequence identities among *SLF* alleles are high and the predicted SLF protein sequences are highly conserved. Based on its sequence similarity with the PavSLFL1 protein, SLF might be considered among the candidates for the general inhibitor role in SI interactions of almond. Further, there are candidate homologs for other PavSLFL-encoding genes near the almond S-locus; their roles are also worthy of investigation. As expected, we observed considerable sequence variation among alleles of the *S-RNase* and *SFB* genes, which are known to encode the determinants of pistil-pollen specificity. The *S-RNase* alleles also vary considerably in length, mainly because of polymorphisms in the second intron.

With analysis of *S-RNase* allele sequences, it was possible to design new marker assays for use in almond breeding. Assays that distinguish *S*_*f*_ alleles from SI alleles can be used to select self-fertile progeny, while those that distinguish among SI alleles can be used to design compatible crosses. Unlike assays that were previously available for some alleles^25–28^, the fluorescence-based KASP assays developed here do not require gel electrophoresis. They can be applied at high throughput to large numbers of samples. Given the considerable advantages of self-fertility in breeding and horticulture, the *S*_*f*_ assays are likely to be particularly useful. Among the *S*_*f*_ assays, the two-primer presence-absence assay WriPdSf-1 should be sufficient for most applications. The others (WriPdSf-2 through WriPdSf-5) could be advantageous in cases where it is useful to know what other allele is present in combination with *S*_*f*_. As all of these assays are based on genomic sequence polymorphisms, none of them can be expected to distinguish the active *S*_*f*_ allele (*S*_*fa*_) from its inactive epi-allele (*S*_*fi*_).

Due to the high level of sequence variation among *S-RNase* alleles, the approaches used for assay design had to go beyond the methods that are routinely used to design KASP assays for individual SNPs within otherwise conserved regions. Some differences could only be detected as presence-absence polymorphisms. Some assays were designed using degenerate primers. Several assays have the same common primer but different sets of alternative allele-specific primers. The approaches used here could be useful for designing markers for other *S*-alleles in almond or other species, or for other multi-allelic loci.

Prior to this research, some variation had been noted in the length and sequence of the interval between the *S-RNase* and *SFB* genes^6,29,30^ and it had been suggested that this variation could contribute to *S*-haplotype specificity by limiting recombination within the interval^26^. Among 11 haplotypes for which this region was completely sequenced, we observed over five-fold variation in length and substantial sequence variation. Consistent with the idea that this could be a region of low recombination, this region is AT-rich. Recombination-enriched sites are often in regions with high GC content^31,32^.

The protein sequences deduced from *S-RNase* allele sequences contain five conserved regions (C1, C2, C3, RC4 and C5) and the hypervariable region (RHV) that are considered characteristic of Rosaceae S-RNases^7,33^, two variable regions (V1 and V2) that had previously been reported between RC4 and C5^26,34^ and three highly variable regions that had not previously been reported. Similarly, the protein sequences deduced from *SFB* allele sequences contain previously reported features (the F-box motif, two variable regions (V1 and V2) and two hypervariable regions (HVa and HVb)^6^ and two additional short highly variable regions (V3 and V4). The Ka/Ks ratio obtained for the complete *S-RNase* allele sequences (0.60) is similar to values that were previously reported for almond^26^, while that obtained for the complete *SFB* allele sequences (0.50) is similar to what has been reported for sweet cherry (*Prunus avium* L.)^35^.

To generate three-dimensional models for almond S-RNases, we needed to select a template from among RNases for which crystal structures had been determined. After thorough evaluation of the sequences of eight candidate RNases in comparison with almond S-RNase sequences, we selected the 1J1G (MC1 RNase) template. This template had previously been used for 3D modelling of three almond S-RNases: the S_8_-, S_23_-, and S_f_-RNases^36^. We constructed models for those three proteins and for two others (the S_5_- and S_7_-RNases). In agreement with what was previously reported^36^, these models consist of α-helices and β-strands that are inter-connected by loops. The variation that we observed in the numbers, lengths and positions of secondary structure elements was similar to that reported for S-RNase proteins of Japanese pear and apple^19,37,38^ but greater than what had been reported for almond^36^. This difference is likely due to the more comprehensive modelling processes used here, with several tools used to align protein sequences and identify mismatches, many models generated from slightly different alignments and final models selected based on optimisation and evaluation of binding energy for multiple stable low-energy models.

Within the selected models, the positions of conserved cysteine residues and the predicted positions of disulphide bridges between them are similar to what has been observed for other members of the T2 RNase enzyme family, including the S_3_- and S_4_-RNases of Japanese pear^19,39^. Disulphide bridges may help stabilise the secondary and tertiary structure of S-RNases, contributing to maintaining the proteins in a flexible yet active conformation^40^.

The active sites of RNases include amino acid residues that temporarily bind with RNA and residues that catalyse cleavage of RNA. Cysteine, histidine, glutamic acid, lysine and tryptophan residues have been proposed to be particularly important for these roles^19,41^. Such residues were observed within the putative active sites that we identified for almond S-RNases, all at positions that correspond with those of similar residues in the active site of the S_3_-RNase of Japanese pear^19^. A conserved cysteine residue in the active site may influence the binding affinity of the protein by enhancing the interaction between the enzyme and its substrate. Conserved histidine residues in the active site may be catalytically important. Histidine residues in the active sites of RNases of the fungus *Rhizopus niveus* M. Yamaz., have been shown to act as the key residues that mediate catalysis^19,42^. Consistent with this, it has been shown that the loss of a histidine residue from the C2 region of an S-RNase leads to self-fertility in Peruvian tomato (*Solanum peruvianum* L.)^43^ and that carboxymethylation of histidine residues inactivates S-RNases in jasmine tobacco (*Nicotiana alata* Link & Otto)^44^. Conserved glutamic acid and lysine residues in the α_5_ element within the active site may be important in stabilising a penta-covalently associated RNA substrate intermediate^42,45^. Conserved tryptophan residues within the active site may be important for fixation of catalytically important histidine and glutamic acid residues^19,42^ via formation of hydrogen bonds between tryptophan residues and the γ-carboxyl groups of glutamic acid residues and/or stacking interactions between the indole ring of tryptophan residues and the imidazole ring of histidine residues. For other RNases, tryptophan residues have been reported to contribute to energy transfer with bound substrates^46,47^. Among the almond S-RNases examined here, two lysine residues are completely conserved: one in C3 and the other between V2 and C5. Another lysine residue, in V2, is conserved among all of the almond S-RNases except S_19_-RNase. The conserved lysine residues in V2 and between V2 and C5 correspond with lysine residues that have been detected in other species of the Rosaceae^38^.

Variation in the numbers, lengths and positions of α-helices, β-sheets and loops may contribute to functional differences among almond S-RNases. Residues at loop-anchoring points could be particularly important in influencing protein folding topologies, depending on their sizes and whether they have hydrophobic/hydrophilic or polar/non-polar characteristics. Among the five proteins that were modelled here, the most obvious structural difference involves an extended loop in the S_f_-RNase. This loop was previously reported, with discussion of how it might contribute to self-fertility^36^. Now that it has been demonstrated that the S-RNase encoded by the *S*_*fa*_ epi-allele can function in self-incompatible interactions^15^, it seems unlikely that the long loop determines self-fertility. Nevertheless, it is intriguing that this S-RNase has such a distinct structural feature.

Although the main-chain backbones of all five almond S-RNases superposed very well with each other in other parts of the molecules, there are noticeable differences in the RHV region, which is exposed on the protein surface. This supports the idea that the RHV region is important in determining specificity. Variation in the length and charge of the loop within RHV could provide flexibility for mediation of intermolecular interactions on the protein surface. The V4 region, which is also on the protein surface may contribute to the regulation of protein-protein interactions by affecting the conformation of secondary structural elements such as α-helices and loops. The hydrophobic and electronegative nature of the α-helices may stabilise the conformation of both the α-helices and the loop.

In the S-RNase proteins examined here, predicted *N-*glycosylation sites were more abundant in internal regions than in C termini and were often within loops and loop anchoring points. *N*-glycosylation of these proteins may be predominantly post-translational rather than co-translational and may contribute to ensuring proper protein folding and stabilising secondary structures such as α-helices and β-sheets. None of these proteins had more than one predicted *O*-glycosylation site, in accordance with previous reports that *O*-glycoslyation is not common in plants^48^.

In *Solanum chacoense*, site-directed mutagenesis of specific sites within the RHV-encoding region of an *S-RNase* allele led to the acquisition of dual SI^49,50^, in which one *S-RNase* can recognise two *SFB* alleles. Here, we applied *in silico* mutagenesis to investigate effects of changing specific residues in the almond S_7_-RNase. High destabilisation energies were observed at N- and C- termini of S-RNase proteins, indicating that these termini are flexible. Stabilising or destabilising effects in the RHV region and differences in the number, types and positions of *N*-glycosylation sites could also contribute to maintaining substrate specificity and function.

This is the first report on high-throughput sequencing of haplotypes of the complex *S*-locus of almond. With this approach, we completed the DNA sequence of the *S*_7_ haplotype and generated complete sequences for three other haplotypes and partial sequences for 11 haplotypes. This provided new information on structural variation within the *S* locus and on allelic variation in the genes that determine SI specificity and made it possible to design high-throughput marker assays for application in almond breeding. The 3D protein modelling, surface morphology assessments and assessments of sites with *N*-glycosylation and/or *O*-glycosylation potential conducted here broaden knowledge on the structure and possible mechanisms of S-RNase-based SI, indicating how numbers, lengths, sequences and positions of secondary structural elements, electrostatic potential and surface conformation of the RHV region and post-translational modification could affect S-RNase function and specificity.

## Methods

### Plant materials, library preparation and DNA sequencing

To enable design of primer pairs that would provide overlapping amplicons from the *S*-locus, the *S*_7_ haplotype sequence (AB081587) was aligned with available sequences for almond *SLF, S-RNase* and *SFB* alleles and for pseudomolecule 6 of peach (*Prunus persica* (L.) Batsch)^51^ (Supplementary Table S17), using ten iterations of the Map to Reference alignment algorithm in Geneious software version 9.0.2^52^. Primer pairs (Supplementary Tables S18 and S19) were designed using Primer3 software (http:/primer3.ut.ee)^53,54^.

Genomic DNA was extracted from young leaves of 48 almond clones (Supplementary Table S1), using an Isolate II Plant DNA Extraction Kit (Bioline, NSW, Australia). DNA quality and quantity were assessed on 1% (w/v) agarose gels using a HyperLadder I DNA ladder (Bioline, NSW, Australia). PCR amplification was performed in a total volume of 20 uL using 20 ng of DNA with 1x Phusion^®^HF, 1.25 mM dNTPs, 1 μM primer mix, and 0.2 U of Phusion High-Fidelity DNA polymerase (New England Biolabs, Ipswich, MA, USA). The PCR conditions used were 98 °C for 30 s, 34 cycles of 98 °C for 10 s, annealing temperature for 30 s and 72 °C for 10 min followed by a final extension at 72 °C for 15 min. Samples (5 μL) of the amplified products and the HyperLadder I DNA ladder (Bioline, NSW, Australia) were run on 1% (w/v) agarose gels at 100 V for 30 min. Gels were stained with SYBR^®^ Safe (Invitrogen, NSW, Australia).

With each of seven primer pairs (Supplementary Table S18), two products differing in length were amplified from each of eight clones that carry the *S*_7_ haplotype (Supplementary Table S19). In each case, one of these products was of the length expected for the *S*_7_ haplotype. When the same primer pairs were applied to clones that do not carry the *S*_7_ haplotype, only four of the seven pairs amplified products. With additional primer pairs (Supplementary Table S20), additional products were amplified (Supplementary Table S21).

Each PCR product was classified as strong or weak based on the intensity of electrophoretic bands. For each almond clone, strong and weak products were pooled in separate tubes. Each pooled sample was purified using AMPure® XP beads (Agencourt Bioscience, MA, USA). For each almond clone, the strong and weak pools were mixed together at a ratio that should provide approximately uniform coverage across the *S* locus.

A sequencing library was prepared using an Illumina Nextera DNA Library Prep Kit (V3) (Illumina, VIC, Australia) and 50 ng of DNA from each of the resulting samples. The Tn5 transposase from the kit was used to digest DNA samples to generate segments of about 300 bp containing read 1 (5′-TCGTCGGCAGCGT-3′) and read 2 (5′-GTCTCGTGGGCTCGG-3′) sequences. Index primers i5 and i7 and paired-end primers P5 and P7 were annealed to each sample using reduced-cycle PCR amplification. Amplified products were purified using AMPure® XP, quantified by qPCR using Kapa SYBR FAST Master Mix (Kapa Biosystems, MA, USA) on a Rotor-Gene Q instrument (QIAGEN, VIC, Australia) and assayed for quality using a TapeStation 2002 instrument (Agilent Technologies, VIC, Australia). Each sample was normalised to 4 nM and the samples were pooled. The resulting library was assessed for quality in a Bioanalyzer 2001 instrument (Agilent Technologies, VIC, Australia), diluted to 12 pM and mixed with 1% (w/v) Illumina PhiX library. Paired-end sequencing was conducted on an Illumina MiSeq instrument, using an Illumina 600-cycle Version 3 reagent kit.

### Sequence analysis

Raw sequence reads were assessed for quality, adapter sequences and barcode contamination using FASTQC v0.11.5 (http://bioinformatics.babraham.ac.uk/projects/fastqc). Adapter sequences were removed using the ILLUMINACLIP option in Trimmomatic V0.32^55^. This was followed by another run of FASTQC. Sequence data were deposited in the National Center for Biotechnology Information (NCBI) Short Read Archive (SRA) as study SRP133723. Trimmed reads from ten clones known to carry the *S*_7_ haplotype were aligned to the AB081587 sequence using the BWA-mem algorithm in the Burrows-Wheeler alignment (BWA-0.6) tool^56^. The resulting binary alignment/map (BAM) files were visualised using Tablet graphical viewer version 1.16.09.06^57^.

Trimmed reads from each clone were assembled using Mimicking Intelligent Read Assembler (MIRA) version 4.0.2^58^. The resulting contig sequences were mapped to the AB081587 sequence and visualised using CONTIGuator software^59^. Further, large contigs (size ≥ 500 bp; N ≥ 50) were aligned to the AB081587 sequence using the Map to Reference function in Geneious software version 9.0.2. Sequences were obtained from output files of the pileup command in SAMtools version 1.2^60^. Polymorphisms were graphically visualised using Integrated Genomic Viewer version 2.3^61^.

Sequences from each of ten clones that carry the *S*_7_ haplotype were aligned with the AB081587 sequence using the Clustal W multiple sequence alignment algorithm^62^ in Geneious software version 9.0.2. An *S*_7_ sequence was established for each clone and an overall consensus sequence was established for *S*_7_. Sequences from *S*_1_*S*_7_, *S*_7_*S*_8_ and *S*_7_*S*_*f*_ clones were then compared to the overall consensus *S*_7_ sequence using the variant call format in VCFtools v0.1.13^63^. This provided haplotype sequences for *S*_1_, *S*_8_, and *S*_*f*_, which were then used as references to obtain partial sequences for other haplotypes. Sequences of *S* haplotypes and *SLF*, *S-RNase* and *SFB* alleles were deposited in GenBank; their accession numbers are listed in Table S2.

Pairwise sequence differences among haplotypes and among *SLF*, *S-RNase*, and *SFB* alleles were determined using Clustal W. Conserved blocks were identified using the Gblocks version 0.91b^64^ tool on the Phylogeny.fr online server (www.phylogeny.fr) with the ‘less stringent’ data selection setting.

Protein-coding sequences of the *S*_1_, *S*_7_, *S*_8_, and *S*_*f*_ haplotypes were predicted and analysed with BLASTX 2.8.^65^ using the refseq protein database, and with GENSCAN^66^ using Arabidopsis (*Arabidopsis thaliana* L.) as the reference. LTR retrotransposons were detected with LTR_Finder version 1.0.5^67^ using an Arabidopsis tRNA database (http://lowelab.ucsc.edu/GtRNAdb/) to predict protein-binding sites. The deduced amino acid sequences of almond SLF proteins were compared to those of sweet cherry SLF-like proteins (XP_021802052.1 (PavSLFL1), XP_021803309.1 (PavSLFL2), XP_021816935 (PavSLFL3), X_P021800841 (PavSLFL4/5), XP_021821224.1 (PavSLFL6), XP_021802446.1 (PavSLFL7) and XP021816963.1 **(**PavSLFL8)^10^. Presence of SLF-like genes in the vicinity of the almond *S*-locus were identified by conducting a homology search using BlastP (word size 6 and an E-value of 1e^−5^) with the query sequences of *P. avium* SLFLs using the genome databases of *P. dulcis* Texas Genome v2.0^68^ in the GDR database (https://www.rosaceae.org/). Coding sequences of *S-RNase* and *SFB* genes were analysed for non-synonymous (Ka) and synonymous (Ks) variation using DnaSP v6.10^69^ with a 100 bp sliding window and 20 bp steps. Regions with sequence identity below 35% were considered to be variable.

### Design and application of S-allele markers

Primer sets consisting of two primers (an allele-specific primer for a target allele and a second primer) or three primers (two allele-specific primers and a common primer) were designed using the *S-RNase* allele sequences following KASP™ (LGC Ltd, Teddington, UK) primer design guidelines^70^ and using Primer 3 software version 4.0^54^. Tail sequences complementary to the FRET cassettes in the KASP Master Mix were added to the 5ʹ ends of the allele-specific primers. The resulting primer sets were named with the prefix WriPdS with Wri referring to the Waite Research Institute, Pd referring to *Prunus dulcis* and S referring to the *S* locus, followed by a number or letter designating a target *S* allele (e.g. f for *S*_*f*_). In cases where more than one primer set was developed to detect the same target allele, a number was appended to distinguish the primer sets (e.g., WriPdS7-1 and WriPdS7-2). Two DNA samples of each of Nonpareil, Antoñeta, Carmel, Francolí, Johnston’s Prolific, Lauranne, Mandaline, Somerton, Vairo, 12–350, Capella, Mira, Carina and Maxima and two water samples (negative controls) were assayed with all primer sets. DNA samples of 10 ng (5 µL of 2 ng/µL) were dried at 55 °C for 1 h. A mixture of 0.028 µL (containing 12 µM of each allele specific forward primer and 30 µM of the common primer) and 1.972 µL of 1 × KASP Master Mix was added to each sample. Amplification was conducted using the standard KASP PCR protocol in a Hydrocycler-^16^ thermocycler (LGC Ltd, Teddington, UK). Fluorescence detection was performed in a Pherastar Plus plate reader (BMG LABTECH, Germany). Each primer set that was shown to be informative based on results from this panel was assayed on progeny of relevant crosses (Supplementary Table S22). For further evaluation of four primer sets, gBlocks® Gene Fragments (Integrated DNA Technologies, Iowa, USA) were synthesised to represent segments of the *S-RNase* alleles *S*_*f*_*, S*_1_, *S*_3_, *S*_5_, *S*_7_, *S*_8_, *S*_9_, *S*_23_ and *S*_25_ (Supplementary Fig. S13). Primer sets WriPdSf-2 through WriPdSf-5 were applied to samples of these synthetic DNA fragments and to 1:1 mixtures of each possible pairwise combination of these fragments (representing heterozygous genotypes) using the KASP screening procedure as described above.

### Analysis of marker data

Fluorescence data were analysed using Kraken™ software (LGC Ltd, Teddington, UK), which normalises FAM and HEX fluorescence intensities relative to an internal ROX control, identifies clusters of data points and assigns individual data points to clusters. Statistical analyses were conducted using R (https://www.R-project.org/). Normalised FAM and HEX fluorescence intensities were subjected to one-way multivariate analysis of variance (MANOVA) with cluster (genotype call) as the independent variable. In cases with more than two clusters, *post hoc* comparisons among clusters were conducted using pairwise Tukey contrasts, as implemented in the R package MANOVA.RM^71^. For molecular marker data collected from the progeny of crosses, chi-square tests were used to assess the deviation of observed genotypic ratios from expected ratios.

### 3D modelling of S-RNase proteins

Protein sequences and crystal structures of eight RNases (1J1G, 1J1F, 1BK7, 1UCA, 1UCC, 1UCD, IUCG, 1V9H) were downloaded from the Protein Data Bank^72^. The sequences were aligned with the deduced protein sequences for almond *S-RNase* alleles using PSI-BLAST^73^, PSIPRED v3.3^74^ and RaptorX^75^.

Five almond S-RNases (S_f_, S_5_, S_7_, S_8_, and S_23_) were subjected to comparative protein modelling using Modeller V9.19^76^. From among known 3D structures with more than 35% sequence identity to the target sequences, the one with the highest positional sequence identity and the lowest E-value was selected for each target S-RNase. Each target S-RNase was aligned with its selected template using MUSCLE alignment^77^ in Geneious version 9.0.2. Alignments were checked using PSIPRED v3.3. For each S-RNase, four structurally aligned sequences were used to construct 100 3D models using Modeller V9.19. From among these models, five models with favourable modeller objective function (MOF)^78^ and discrete optimised protein energy (DOPE)^79^ parameters were selected. Each model was optimised using FoldX4^80^ and evaluated using ProSa 2003^81^ and PROCHECK^82^. Energy and stability were calculated using FoldX4. Based on all evaluations, the best-scoring models were selected for each S-RNase. Selected models were superposed on the template structures, yielding root mean square deviation (RMSD) values as indicators of structural folds. Images were generated with PyMOL Molecular Graphics V1.8.2.0 (Schrödinger LLC, NY, USA).

To identify putative catalytic sites, PSI-BLAST, PSIPRED and PROMALS3D were used to align almond S-RNase sequences with the sequence of the S_3_-RNase of Japanese pear (*Pyrus pyrifolia* (Burm.) Nak) (BAA93052.1)^83^. The positions of disulphide bonds in the S-RNases were identified by using the SSBOND record of PDB files of ribonucleases and using protein sequences as input in the Disulfind online server (http://disulfind.dsi.unif.it/process.php). Molecular surfaces of S-RNases were generated in PyMOL, using a probe radius of 1.80 Å. Solvent-accessible areas were estimated with Naccess V2.1.1 (http://wolf.bms.umist.ac.uk/naccess/). Electrostatic potentials were calculated with the Adaptive Poisson-Boltzmann Solver^84^ using the PyMOL plug-in APBS Tool2, with AMBER force field parameters^85^ and dielectric constants of 78 (solvent) and 2 (solute). The values of electrostatic potentials were expressed using Boltzmann constant (k) and the temperature (T) per Einstein (e) (kT/e).

Amino-acid residue positions were investigated for their potential to create new S-RNase specificities by examining Ka/Ks ratios, electrostatic potentials and the cumulative charges of the residues in positively charged regions.

The sequences of five S-RNases (S_5_, S_7_, S_8_, S_23_ and S_f_) were aligned using PROMALS3D^86^ (Supplementary Fig. S12) to identify positions at which the S_7_-RNase sequence differed from the sequence(s) of one or more of the other four S-RNases. At each of these positions, the BuildModel command in FoldX4^80^ was used to mutate the S_7_-RNase residue to the alternative residue (or residues), considering both single and multiple mutation options. A similar process was applied to the S_8_-RNase, with residues replaced by those in the S_7_-RNase. The energies for the wild-type (ΔG wild-type) and mutant (ΔG mutant) proteins were computed using FoldX4) to find differences in stability (ΔΔG = ΔG mutant −ΔG wild-type) of proteins. The ΔΔG values were classified into seven bins based on the standard deviation in FoldX: (i) highly stabilising (ΔΔG < −1.84 kcal/mol), (ii) stabilising (−1.84 kcal/mol ≤ ΔΔG < −0.92 kcal/mol), (iii) slightly stabilising (−0.92 kcal/mol ≤ΔΔG < −0.46 kcal/mol), (iv) neutral (−0.46 kcal/mol <ΔΔG ≤ +0.46 kcal/mol), (v) slightly destabilising (+0.46 kcal/mol <ΔΔG ≤ +0.92 kcal/mol), (vi) destabilising (+0.92 kcal/mol <ΔΔG ≤ +1.84 kcal/mol), and (vii) highly destabilising (ΔΔG > +1.84 kcal/mol).

The sequences of five S-RNases (S_5_, S_7_, S_8_, S_23_ and S_f_) were analysed using the NetNGlyc 1.0 server (http://www.dtu.dk/services/NetNGlyc/) and sequons with *N*-glycosylation potential greater than 0.5 were selected as potential *N*-glycosylation sites. Within the sequons identified as potential *N*-glycosylation sites in the S_7_-RNase, Asn and Xaa residues were replaced by the corresponding residues from the S_8_-RNase, using the BuildModel command of FoldX4^80^. *O*-glycosylation sites in the same five S-RNases were identified using the DictyOGlyc 1.1 Server (http://www.cbs.dtu.dk/services/DictyOGlyc/), with sites with *O*-glycosylation potential greater than 0.5 considered as potential *O*-glycosylation sites.

## Supplementary information

Supplementary  Information

## Data Availability

Sequence data have been deposited in the National Center for Biotechnology information (NCBI) Short Read Archive (SRA) (https://www.ncbi.nlm.nih.gov/sra/) as study SRP133723. *S*-haplotype sequences and allele sequences for *SFB*, *S-RNase* and *SFB* genes have been deposited in the National Center for Biotechnology information (NCBI) GenBank database (https://www.ncbi.nlm.nih.gov/genbank/). GenBank accession numbers are listed in Supplementary Table S2.
