# Supplementary material for: Variation among S-locus haplotypes and among stylar RNases in almond
Source: Sci Rep. 2020 Jan 17;10:583. doi: 10.1038/s41598-020-57498-6 (PMC6969032; doi:10.1038/s41598-020-57498-6)
Supplement: Supplementary file 1 — Supplementary Information [file 41598_2020_57498_MOESM1_ESM.pdf]

Supplementary Tables and Figures

Variation among *S*-locus haplotypes and among stylar RNases in almond

Shashi N. Goonetilleke[1], Adam E. Croxford[1], Timothy J. March[1], Michelle G. Wirthensohn[1], Maria Hrmova[1,2] and Diane E. Mather[1]*

[1]School of Agriculture, Food and Wine, Waite Research Institute, The University of Adelaide, PMB 1, Glen Osmond, SA 5064, Australia

[2]School of Life Sciences, Huaiyin Normal University, Huai'an 223300, China

***Corresponding author:** diane.mather@adelaide.edu.au; +61 8 8313 7156; +61 420 959 955

Table S1
Almond cultivars and breeding selections used in this research

Cultivar or breeding selection	Origin	*S* genotype	Reference
Antoñeta	Spain	S_1S_f	Dicenta F, Ortega E, Cánovas J A, Egea J. Self-pollination vs. cross-pollination in almond: pollen tube growth, fruit set and fruit characteristics. Plant Breed. **121,**163-167 (2002).
Atkinson's Hardshell	Australia	$S_{14}S_{27}$	Wirthensohn M, Rahemi M, Fernández i Martí A,Identification of self-incompatibility genotypes and DNA fingerprinting of some Australian almond cultivars. Acta Hortic. **912,** 561-566 (2011).
Baxendale	Australia	S_5S_7	Channuntapipat C, Wirthensohn M, Ramesh S, Batlle I, Arus P, Sedgley M, Collins G. Identification of incompatibility genotypes in almond (*Prunus dulcis* Mill.) using specific primers based on the introns of the *S*-alleles. Plant Breed. **122,** 164 -168 (2003).
Biggs Hardshell	Australia	S_6S_{14}	Wirthensohn M, Rahemi M, Fernández i Martí A. Identification of self-incompatibility genotypes and DNA fingerprinting of some Australian almond cultivars. Acta Hortic. **912**, 561-566 (2011).
Brown Nonpareil	Australia	S_1S_7	Wirthensohn M, Rahemi M, Fernández i Martí A (2011) Identification of self-incompatibility genotypes and DNA fingerprinting of some Australian almond cultivars. Acta Hortic. **912,** 561-566 (2011).
Brown Brandis	Australia	$S_{23}S_{25}$	Wirthensohn M, Rahemi M, Fernández i Martí A (2011) Identification of self-incompatibility genotypes and DNA fingerprinting of some Australian almond cultivars. Acta Hortic. **912,** 561-566 (2011).
Bruce	Australia	$S_{22}S_{23}$	Wirthensohn M, Rahemi M, Fernández i Martí A (2011) Identification of self-incompatibility genotypes and DNA fingerprinting of some Australian almond cultivars. Acta Hortic. **912,** 561-566 (2011).
Capella	Australia	S_7S_f	Plant Varieties Journal **29**, 41 (2016).
Carina	Australia	S_7S_f	Plant Varieties Journal **29**, 42 (2016).
Carmel	USA	S_5S_8	Bošković R, Tobutt KR, Batlle I, Duval H, Martinez-Gomez P, Gradziel TM. Stylar ribonucleases in almond: correlation with and prediction of incompatibility genotypes. Plant Breed **122,** 1; 70-76 (2003).
Chellaston	Australia	Ambiguous	Channuntapipat C, Wirthensohn M, Ramesh S, Batlle I, Arus P, Sedgley M, Collins G. Identification of incompatibility genotypes in almond (*Prunus dulcis* Mill.) using specific primers based on the introns of the *S*-alleles. Plant Breed **122,** 164 -168 (2003).
Clements	Australia	S_6S_{14}	Unpublished data
Constantí	Spain	S_3S_f	Vargas F, Romero M, Clavé J, Vergés J, Santos J, Batlle I. 'Vayro','Marinada','Constantí', and 'Tarraco'Almonds. HortSci **43**, 535-537 (2008).
Federation	Australia	S_3S_{22}	Unpublished data
Francolí	Spain	S_1S_f	Martínez-Gómez P, Dandekar A, Gradziel T, Alonso J, Lopez M, Batlle I, Ortega E, Sanchez-Perez R, Dicenta F. Identification of self-incompatibility alleles in almond and related Prunus species using PCR. Acta Hortic **622,** 397-402 (2003).
Frenzy	Australia	S_5S_8	Wirthensohn M, Rahemi M, Fernández i Martí A. Identification of self-incompatibility genotypes and DNA fingerprinting of some Australian almond cultivars. Acta Hortic **912,** 561-566 (2011).
Johnston's Prolific	Australia	$S_{23}S_{25}$	Wirthensohn M, Rahemi M, Fernández i Martí A. Identification of self-incompatibility genotypes and DNA fingerprinting of some Australian almond cultivars. Acta Hortic **912,** 561-566 (2011).
Jordan	Spain	S_8S_{23}	Unpublished data
Keanes	Australia	S_7S_{27}	Wirthensohn M, Rahemi M, Fernández i Martí A. Identification of self-incompatibility genotypes and DNA fingerprinting of some Australian almond cultivars. Acta Hortic **912,** 561-566 (2011).
Kapareil	USA	S_8S_{13}	Kester D. Kapareil - A new small-kernel almond variety for confections. Calif Agr **16**(2), 10-11 (1962).
Lauranne	France	S_3S_f	Bošković R, Tobutt KR, Batlle I, Duval H. Correlation of ribonuclease zymograms and incompatibility genotypes in almond. Euphytica **97**, 167-176 (1997).
LeGrand	USA	S_1S_8	Channuntapipat C, Wirthensohn M, Ramesh S, Batlle I, Arus P, Sedgley M, Collins G. Identification of incompatibility genotypes in almond (*Prunus dulcis* Mill.) using specific primers based on the introns of the *S*-alleles. Plant Breed **122,** 164 -168 (2003).
Mandaline	France	S_1S_f	Duval, H. (1999). 'Mandaline', a new French almond variety. FAO-CIHEAM Nucis Newsletter, **8**: 36.
Marion Sturt Creek	Australia	S_1S_{22}	Wirthensohn M, Rahemi M, Fernández i Martí A. Identification of self-incompatibility genotypes and DNA fingerprinting of some Australian almond cultivars. Acta Hortic **912,** 561-566 (2011).
Maxima	Australia	S_3S_8	Plant Varieties Journal **29**(1), 43 (2016).
McKinlays	Australia	S_7S_8	Channuntapipat C, Wirthensohn M, Ramesh S, Batlle I, Arus P, Sedgley M, Collins G. Identification of incompatibility genotypes in almond (*Prunus dulcis* Mill.) using specific primers based on the introns of the *S*-alleles. Plant Breed. **122,** 164 -168 (2003).
Milow	USA	S_1S_8	Channuntapipat C, Wirthensohn M, Ramesh S, Batlle I, Arus P, Sedgley M, Collins G (2003) Identification of incompatibility genotypes in almond (*Prunus dulcis* Mill.) using specific primers based on the introns of the *S*-alleles. Plant Breed **122**:164 -168
Mira	Australia	S_7S_f	Plant Varieties Journal **29**, 40 (2016).
Monarto 2	Australia	S_8S_{25}	Wirthensohn M, Rahemi M, Fernández i Martí A. Identification of self-incompatibility genotypes and DNA fingerprinting of some Australian almond cultivars. Acta Hortic **912,** 561-566 (2011).
Monarto 3	Australia	Unknown	Wirthensohn M, Rahemi M, Fernández i Martí A. Identification of self-incompatibility genotypes and DNA fingerprinting of some Australian almond cultivars. Acta Hortic **912,** 561-566 (2011).
Nonpareil	USA	S_7S_8	Bošković R, Tobutt KR, Batlle I, Duval H. Correlation of ribonuclease zymograms and incompatibility genotypes in almond. Euphytica **97**, 167-176 (1997).
Parkinson	Australia	$S_{22}S_{23}$	Wirthensohn M, Rahemi M, Fernández i Martí A. Identification of self-incompatibility genotypes and DNA fingerprinting of some Australian almond cultivars. Acta Hortic **912,** 561-566 (2011).
Pethick Wonder	Australia	$S_{23}S_{27}$	Wirthensohn M, Rahemi M, Fernández i Martí A. Identification of self-incompatibility genotypes and DNA fingerprinting of some Australian almond cultivars. Acta Hortic **912,** 561-566 (2011).
Pearce	Australia	Unknown	Channuntapipat C, Wirthensohn M, Ramesh S, Batlle I, Arus P, Sedgley M, Collins G. Identification of incompatibility genotypes in almond (*Prunus dulcis* Mill.) using specific primers based on the introns of the S-alleles. Plant Breed **122**, 164 -168 (2003).
Peerless	USA	S_1S_6	Bošković R, Tobutt KR, Batlle I, Duval H, Martinez-Gomez P, Gradziel TM. Stylar ribonucleases in almond: correlation with and prediction of incompatibility genotypes. Plant Breed **122**, 70-76 (2003).

Table S1, continued

Cultivar or breeding selection	Origin	*S* genotype	Reference
Ramillete	Spain	S_6S_{23}	Vargas, F.J.; R. Morán (editors). Variedades tipificadas de almendra en España (in Spanish). Monografies de l'Obra Agrícola de la Caixa de Pensions. 77 (Fundació Caixa de Pensions, 1984).
Softshell Jordan	Australia	S_8S_{14}	Unpublished data
Somerton	Australia	S_1S_{23}	Channuntapipat C, Wirthensohn M, Ramesh S, Batlle I, Arus P, Sedgley M, Collins G. Identification of incompatibility genotypes in almond (*Prunus dulcis* Mill.) using specific primers based on the introns of the *S*-alleles. Plant Breed **122**, 164 -168 (2003).
Steliette	France	Ambiguous	Bošković R, Tobutt KR, Batlle I, Duval H. Correlation of ribonuclease zymograms and incompatibility genotypes in almond. Euphytica **97**, 167-176 (1997).
Strout's Papershell	Australia	$S_{22}S_{25}$	Wirthensohn M, Rahemi M, Fernández i Martí A. Identification of self-incompatibility genotypes and DNA fingerprinting of some Australian almond cultivars. Acta Hortic **912,** 561-566 (2011).
Tom Strout	Australia	Unknown	Wirthensohn M, Rahemi M, Fernández i Martí A. Identification of self-incompatibility genotypes and DNA fingerprinting of some Australian almond cultivars. Acta Hortic **912,** 561-566 (2011).
Vairo	Spain	S_9S_f	Vargas F, Romero M, Clavé J, Vergés J, Santos J, Batlle I. 'Vayro','Marinada','Constantí', and 'Tarraco'Almonds. HortSci **43**, 535-537 (2008).
White Brandis	Australia	S_6S_{23}	Wirthensohn M, Rahemi M, Fernández i Martí A. Identification of self-incompatibility genotypes and DNA fingerprinting of some Australian almond cultivars. Acta Hortic **912,** 561-566 (2011).
12-350	Spain	S_1S_f	Batlle I, Ballester J, Romero MA, Vargas FJ. Use of stylar ribonucleases in almond breeding to design crosses and select self-compatible seedlings. Nucis **6,** 12-14 (1997).
T5	Australia	S_7S_f	Unpublished data
T6	Australia	S_8S_f	Unpublished data
T7	Australia	S_7S_f	Unpublished data
T8	Australia	Unknown	Unpublished data

Table S2
GenBank accession numbers

Haplotype	Source	*S*-locus haplotype	*SLF* allele	*S-RNase* allele	*SFB* allele
S_7	Nonpareil	MH029536	MH316060	MH316080	MH316104
S_7	Mira	MH029537	MH316072	MH316081	MH316097
S_7	McKinlays	MH029538			
S_7	Keanes	MH029539		MH316076	
S_7	Capella	MH029540		MH316075	MH316100
S_7	Brown Nonpareil	MH029541			
S_7	T5	MH029542			
S_7	T7	MH029543			
S_7	Carina	MH029544			
S_7	Baxendale	MH029545			
S_f	Mira	MH029546	MH316074	MH316090	MH316111
S_f	Carina	MH029547	MH316070	MH316088	MH316099
S_f	Capella	MH029548			
S_f	T5	MH029549			
S_f	T7	MH029550			
S_8	Nonpareil	MH064152	MH316073	MH316089	MH316105
S_8	McKinlays	MH064153	MH316061	MH316082	MH316098
S_1	Brown Nonpareil	MH064154	MH316056	MH316077	MH316096
S_{13}	Kapareil	MH064155	MH316063	MH316083	MH316107
S_{14}	Biggs Hardshell	MH064156	MH316064	MH316084	MH316108
S_{19}	Tom Strout	MH064157	MH316065	MH316085	MH316102
S_{22}	Strout's Papershell	MH064158	MH316066	MH316091	MH316109
S_{23}	Chellaston	MH064159	MH316067	MH316086	MH348868
S_{25}	Johnston’s Prolific	MH064160	MH316068	MH316087	MH316110
S_{27}	Keanes	MH064161	MH316069	MH316093	MH316101
S_3	Constantí	MH064162	MH316071	MH316095	MH348866
S_3	Lauranne	MH064163	MH316057	MH316094	MH316112
S_5	Carmel	MH064164	MH316058	MH316078	MH348867
S_6	Ramillete	MH064165	MH316059	MH316079	MH316103
S_9	Vairo	MH064166	MH316062	MH316092	MH316106

Table S3
Percentage nucleotide identity among four almond *S* haplotypes

***S* haplotype**	S_1	S_7	S_8
S_7	79		
S_8	83	84	
S_f	60	52	51

Table S4

Open reading frames (ORFS) predicted by GENSCAN and a summary of BLASTX homology search results

**Haplotype**	**ORF**	**Position in *S*-locus (bp)**	**Homology to known protein**	**Protein accession number**	**Protein description**	**Identity (%)**	**E-value**
S_1	ORF1	40-1,029	SLF$_1$ (ORF1)	AAZ15104.1	F -box protein	90	1.00E-178
	ORF2	1,029-1,533	SLF$_1$ (exons 2.0 and 2.1 of ORF2)	-	-	-	-
		3,178- 4,032	Uncharacterised protein LOC107881795 [*Prunus mume*] (exons 2.3 and 2.4 of ORF2)	XP_016652015.1	Unknown protein	85	1.00E-178
	ORF3	7,593-9,037	S$_1$-RNASE (ORF3)	BAA34663.1	S-RNase	95	0
	ORF4	9,289-10,533	SFB$_1$ (ORF4)	BAC65207.1	SFB	96	0
	ORF5	10,566-13,840	SFB$_1$ (ORF5)	-	-	96	0
	ORF6	-	-	-	-	-	-
	ORF7	-	-	-	-	-	-
	ORF8	-	-	-	-	-	-
	ORF9	-	-	-	-	-	-
	ORF10	-	-	-	-	-	-
	ORF11	30,197-33,800	Uncharacterised protein LOC110760030 [*Prunus avium*] (ORF11)	XP_021817896	DDE superfamily endonuclease; cl21562	69	2.00E-118
	ORF12	34,285-42,279	Uncharacterised mitochondrial protein AtMg00810-like [*Ziziphus jujuba*] (ORF12)	XP_015883892	Reverse transcriptase (RNA-dependent DNA polymerase	68	1.00E-118
	ORF13	45,996-51,080	Uncharacterised mitochondrial protein AtMg00810-like [*Ziziphus jujuba*] (ORF13)	XP_015883893	Reverse transcriptase (RNA-dependent DNA polymerase	68	1.00E-118
	ORF14	51,336-53,542	Uncharacterised protein LOC107024343 *[Solanum pennellii]* (ORF14)	XP_015080794	Uncharacterised protein LOC107024343	0	5.60E+01
	ORF15	65,650-67,251	Uncharacterised protein LOC108175069 [*Malus domestica*] (ORF15)	XP_017192360	Uncharacterised protein LOC108175069	78	1.00E-175
	ORF16	-	-	-		-	-
S_7	ORF1	30-1,702	SLF$_7$ (exons 1.0 and 1.1 of ORF1)	BAC65202.1	F -box protein	100	-
		8,220- 9,110	S$_7$-RNASE (exons 1.9 and 1.10 of ORF1)	BAC65203.1	S-RNase	100	-
	ORF2	9,111-10,185	S$_7$-RNASE (ORF2)	-	S-RNase	100	-
	ORF3	10,243-11,983	SFB$_7$ (ORF3)	BAC65204.1	SFB	-	-
	ORF4	-	-	-	-	-	-
	ORF5	-	-	-	-	-	-
	ORF6	26,746-27,106	Uncharacterised protein LOC110744978 [*Prunus avium*]	XP_021800718.1	Transposase protein necessary for efficient DNA transposition	92	3.00E-174
	ORF7	27,189-27,780					
	ORF8	32,110-33,500	Uncharacterised protein LOC103337099 [*Prunus mume*]	XP_008238475.1	Plant transposon protein	88	1.00E-175
	ORF9	-		-	-	-	-
	ORF10	38,435-41,099	Uncharacterised protein LOC107024343 [*Solanum pennellii*]	XP_015080794.1	Transposase InsO -pfam07727	55	2.00E-173
	ORF11	51,612-52,302					
	ORF12	-	-	-	-	-	-
S_8	ORF1	75- 1,874	F-box/kelch-repeat protein At3g06240-like [*Prunus mume*] (exons 1 and 2 of ORF1)	XP_008245489.1	F-box protein	80	1.00E-150
		7,816 -10,563	S$_8$-RNase (exons 11-14 of ORF1)	BAI70445.1	S-RNase	-	-
	ORF2	10,563-10,644	S$_8$-RNase (ORF2)	-	S-RNase	-	-
	ORF3	13,163-15,260	SFB$_8$ (ORF3)	ACI43065.1	-	-	-
	ORF4	15,848 - 23,608	Protein ALP1-like [*Prunus avium*] (ORF4)	XP_021809190	Plant transposon protein; pfam04827	92	0
	ORF5	23,103- 26,013	Protein ALP1-like [*Prunus avium*] (ORF5)	XP_021811714	Transposon protein; pfam04827	79	0
	ORF6	26,040 - 27,639	Putative nuclease HARBI1 [*Prunus mume*] (ORF6)	XP_016650419	Putative nuclease HARBI1	83	0
	ORF7	30,222-33,349	Uncharacterised protein LOC110744978 [*Prunus avium*] (ORF7)	XP_021800718.1	Uncharacterised protein LOC110744978	83	0
	ORF8	34,209-42,574	Uncharacterised protein BNAC09G10640D [*Brassica napus*] (ORF8)	XP_013746494	Gag-polypeptide of LTR copia-type; pfam14223	64	0
	ORF9	42,644-46,076	Uncharacterised protein BNAC09G10640D [*Brassica napus*] (ORF9)	XP_013746495	Gag-polypeptide of LTR copia-type; pfam14223	64	0
	ORF10	52,069-63,547	Uncharacterised protein LOC109949598 [*Prunus persica*] (ORF10)	XP_020421259	Reverse transcriptase (RNA-dependent DNA polymerase); pfam07727	80	0

Table S4, continued

Haplotype	ORF	Position in *S*-locus (bp)	Homology to known protein	Protein accession number	Protein description	Identity (%)	E-value
	ORF11	63,605-66,406	Uncharacterised protein LOC108175069 [*Malus domestica*] (ORF11)	XP_017192360	Uncharacterised protein LOC108175069	80	0
	ORF12	-	-	-	-	-	
S_f	ORF1	70-1,765	F-box/kelch-repeat protein At3g06240-like [*Prunus mume*] (exons 1 and 2 of ORF1)	XP_008245489.1	F-box protein	81	1.00E-150
		8,150 - 9,375	S_f-RNase (exons 9-13 of ORF1)	AAL35959.2	S-RNase	100	-
	ORF2	9,375-10,173	S_f-RNase (ORF2)	-	S-RNase	100	-
	ORF3	-	-	-	-	-	-
	ORF4	15,400-18,895	SFB_f (ORF4)	BAI68429.1	SFB	100	-
	ORF5	-	-	-	-	-	-
	ORF6	-	-	-	-	-	-
	ORF7	-	-	-	-	-	-
	ORF8	26,251- 28,172	Protein ALP1-like [*Prunus avium]* (ORF8)	XP_021809190.1	Plant transposon protein; pfam04827	94	1.00E-100
	ORF9	-	-	-	-	-	-
	ORF10	-	-	-		-	-
	ORF11	37,117 - 43,090	Uncharacterised protein LOC107024343 [*Solanum pennellii*] (ORF11)	XP_015080794.1	Ty1/Copia family of RNase H	55	2.00E-125
	ORF12	43, 307- 45,829	Uncharacterised protein LOC107024343 [*Solanum pennellii*] (ORF12)	XP_015080794.1	-	-	-
	ORF13	46,069-51,153	Uncharacterised protein LOC107024343 [*Solanum pennellii*] (ORF13)	XP_015080794.1	-	-	-
	ORF14	51,409- 60,936	Uncharacterised protein LOC107024343 [Solanum pennellii] (ORF14)	XP_015080794.1	-	-	-
	ORF15	61,170 - 62,623	Uncharacterised protein LOC110224828 [*Arabidopsis lyrata* subsp. Lyrata] (ORF15)	XP_020867873	Gag-polypeptide of LTR copia-type; pfam14223	79	1.00E-150
	ORF16	62,381- 64,055	Uncharacterised protein LOC108175069 [*Malus domestica*] (ORF16)	XP_017192360.1	Uncharacterized protein LOC108175069	85	1.00E-175
	ORF17	64,113- 68,330	Uncharacterised protein LOC109948602 [*Prunus persica*] (ORF17)	XM_020562248	Gag-polypeptide of LTR copia-type; pfam14223	79	1.00E-145
	ORF18	-	-	-	-	-	

Table S5
Percentage nucleotide identity in the region between the *S-RNase* and *SFB* genes, among 11 almond *S*-locus haplotypes for which the intergenic region was completely sequenced

***S* allele**	S_1	S_3	S_5	S_7	S_8	S_9	S_{22}	S_{23}	S_{25}	S_{27}
S_3	52									
S_5	60	61								
S_7	68	62	57							
S_8	51	65	58	65						
S_9	55	67	59	64	39					
S_{22}	52	69	62	65	42	51				
S_{23}	56	59	65	98	38	45	51			
S_{25}	59	58	64	67	38	54	78	83		
S_{27}	71	59	63	27	38	43	81	82	59	
S_f	21	39	45	35	33	39	38	38	37	52

Table S6
Percentage nucleotide identity among 15 almond *SLF* alleles

***S* allele**[a]	S_{1}	S_{3}	S_{5}	S_{6}	S_{7}	S_{8}	S_{9}	S_{13}	S_{14}	S_{19}	S_{22}	S_{23}	S_{25}	S_{27}
S_{3}	96													
S_{5}	90	88												
S_{6}	96	95	88											
S_{7}	94	95	94	95										
S_{8}	92	93	85	93	93									
S_{9}	96	94	88	95	95	92								
S_{13}	82	74	81	95	91	71	73							
S_{14}	90	89	85	81	93	88	90	72						
S_{19}	98	97	98	96	93	93	97	97	91					
S_{22}	94	93	92	94	94	93	96	89	92	93				
S_{23}	94	93	87	94	90	89	93	70	88	94	94			
S_{25}	88	83	87	94	96	83	81	73	81	93	93	73		
S_{27}	75	82	85	91	91	79	81	81	78	78	99	79	96	
S_{f}	92	91	84	92	93	91	92	70	81	81	92	92	82	78

[a] GenBank accession numbers of the *SLF* alleles that were used in this analysis: MH316056 (S_{1}), MH316057 (S_{3}), MH316058 (S_{5}), MH316059 (S_{6}), MH316060 (S_{7}), MH316073 (S_{8}), MH316062 (S_{9}), MH316063 (S_{13}), MH316064 (S_{14}), MH316065 (S_{19}), MH316066 (S_{22}), MH316067 (S_{23}), MH316068 (S_{25}), MH316069 (S_{27}), MH316070 (S_{f})

Table S7

Percentage sequence identity between sweet cherry SLF-like proteins and the predicted products of 15 almond *SLF* alleles

**Sweet cherry SLF-like protein**	**GenBank accession number**		**Almond *SLF* allele**													
		SLF_{1}	SLF_{3}	SLF_{5}	SLF_{6}	SLF_{7}	SLF_{8}	SLF_{9}	SLF_{13}	SLF_{14}	SLF_{19}	SLF_{22}	SLF_{23}	SLF_{25}	SLF_{27}	SLF_{f}
PavSLFL1	XP021802052.1	88	93	88	93	95	95	92	91	85	92	89	82	92	91	85
PavSLFL2	XP021803309.1	33	33	33	33	36	36	34	32	32	32	32	32	33	33	33
PavSLFL3	XP021816935.1	45	45	44	45	44	45	44	45	44	44	43	43	44	45	44
PavSLFL4/5	XP021800841.1	58	57	57	58	61	61	58	59	57	57	55	56	56	58	58
PavSLFL6	XP021821224.1	31	30	30	30	33	33	31	31	29	31	31	30	29	31	28
PavSLFL7	XP021802446.1	33	33	33	33	34	34	33	34	32	34	32	32	35	33	33
PavSLFL8	XP021816963.1	33	34	33	33	37	37	34	32	32	34	32	32	33	34	33

Table S8
Percentage nucleotide identity among 11 completely sequenced almond *S-RNase* alleles

***S* allele**[a]	S_1	S_5	S_7	S_8	S_{13}	S_{14}	S_{22}	S_{23}	S_{25}	S_{27}
S_5	36									
S_7	28	34								
S_8	39	29	26							
S_{13}	25	34	45	20						
S_{14}	29	34	46	21	37					
S_{22}	28	27	46	20	41	39				
S_{23}	40	39	29	20	31	25	30			
S_{25}	40	39	30	29	20	31	25	34		
S_{27}	34	24	51	23	49	34	39	33	31	
S_f	37	32	29	27	27	27	22	29	28	25

[a] GenBank accession numbers of the *S-RNase* alleles that were used in this analysis: MH316077 (S_1), MH316078 (S_5), MH316080 (S_7), MH316089 (S_8), MH316083 (S_{13}), MH316084 (S_{14}), MH316091 (S_{22}), MH316086 (S_{23}), MH316087 (S_{25}), MH316093 (S_{27}), MH316090 (S_f)

Table S9
Percentage nucleotide identity among 11 completely sequenced almond *SFB* alleles

***S* allele**[a]	S_1	S_5	S_7	S_8	S_{13}	S_{14}	S_{22}	S_{23}	S_{25}	S_{27}
S_5	79									
S_7	71	81								
S_8	36	39	36							
S_{13}	39	30	29	39						
S_{14}	84	79	80	38	39					
S_{22}	85	49	81	38	42	59				
S_{23}	86	79	81	37	38	42	51			
S_{25}	78	85	76	36	38	54	83	83		
S_{27}	85	59	81	37	38	43	81	82	83	
S_f	31	39	38	35	33	39	38	38	37	40

[a] GenBank accession numbers of the *SFB* alleles that were used in this analysis: MH316096 (S_1), MH316112 (S_3), MH348867 (S_5), MH316104 (S_7), MH316105 (S_8), MH316106 (S_9), MH316109 (S_{22}), MH348868 (S_{23}), MH316110 (S_{25}), MH316101(S_{27}), MH316111 (S_f)

Table S10

Sets of primers designed to distinguish among almond *S-RNase* alleles showing the fluorescence (HEX or FAM) expected for each allele

Primer set	Primer sequences (5′ - 3′)[a]	***S-RNase* alleles**								
		S_1	S_3	S_5	S_7	S_8	S_9	S_{23}	S_{25}	S_f
WriPdSf-1	GAAGGTCGGAGTCAACGG**ATT**AATGCAACTAGTCATGCATTTATTTCATG<br>ACCAGTGTTAAGTTTAAAAGTTAGTGGAAT	-	-	-	-	-	-	-	-	HEX
WriPdSf-2	GAAGGTGACCAAGTTCATGC**T**TGGGTTTGAATAATTACTTGGCCATAT<br>GAAGGTCGGAGTCAACGGA**TT**GGGTTTGAATAATTACTTGGCCATAG<br>CAATTTGTGCAACAATGGCCACC	-	HEX	HEX	-	-	HEX	HEX	HEX	FAM
WriPdSf-3	GAAGGTGACCAAGTTCATGC**T**TGGGTTTGAATARTTACTTGGCCATAT<br>GAAGGTCGGAGTCAACGGA**TT**GGGTTTGAATARTTACTTGGCCATAG<br>CAATTTGTGCAACAATGGCCACC	HEX	HEX	HEX	-	HEX	HEX	HEX	HEX	FAM
WriPdSf-4	GAAGGTGACCAAGTTCATGC**T**TGGGTTTGAAWAATTACTTGGCCATAT<br>GAAGGTCGGAGTCAACGGA**TT**GGGTTTGAAWAATTACTTGGCCATAG<br>CAATTTGTGCAACAATGGCCACC	-	HEX	HEX	HEX	-	HEX	HEX	HEX	FAM
WriPdSf-5	GAAGGTGACCAAGTTCATGC**T**TTKGAATAATTACTTGGCCATAT<br>GAAGGTCGGAGTCAACGGA**TT**TTKGAATAATTACTTGGCCATAG<br>TTTGTGCAACAATGGCCACC	-	HEX	HEX	-	-	HEX	HEX	HEX	FAM
WriPdS1	GAAGGTGACCAAGTTCATGCTGAATGGAACAAACATGGTACATGTTCG<br>CCACATTTCGTGGGATCGCTCGAAG	FAM	-	-	-	-	-	-	-	-
WriPdS3	GAAGGTGACCAAGTTCATGCTTGGTACGATTGAAGCGTTTTTAAGGATC<br>GAAGGTCGGAGTCAACGGATTTGGTACGATTGAAGCGTTTTTAAGGATT<br>GGGAAGGCGAATGGAACAAACATGG	HEX	FAM	-	-	-	-	-	HEX	-
WriPdS5	GAAGGTGACCAAGTTCATGCTTGGATGTTGCAGGCTCCTAAAT<br>ACGTTGGGCCAAGATATCTTCA	-	-	FAM	-	-	-	-	-	-
WriPdS7-1	GAAGGTGACCAAGTTCATGCTCTTGGCCATAKGCCATGGATT<br>GAAGGTCGGAGTCAACGGATTCTTGGCCATAKGCCATGGATG<br>CAATTTGTGCAACAATGGCCACC	HEX	HEX	HEX	FAM	HEX	HEX	HEX	HEX	HEX
WriPdS7-2	GAAGGTGACCAAGTTCATGCTAAATTTTAAATTTTGTAATATGAAAAAGTGTG<br>CATTGGTTAATATAAACATTAAGAATTGAA	-	-	-	FAM	-	-	-	-	-

Table S10, continued

Primer set	Primer sequences (5′ - 3′)[a]	*S-RNase* alleles								
		S_1	S_3	S_5	S_7	S_8	S_9	S_{23}	S_{25}	S_f
WriPdS8	GAAGGTGACCAAGTTCATGCTGTTTTGGGAAGGCGAATGGAACAAG<br>CGTCTTTAAGGATATTTGTAATATTGTACGACC	-	-	-	-	FAM	-	-	-	-
WriPdS9	GAAGGTGACCAAGTTCATGCTTTGTGCGAGTACCACATGTCTTGC<br>GAATGGAACAAACATGGTACATGTTCCG	-	-	-	-	-	FAM	-	-	-
WriPdS23	GAAGGTGACCAAGTTCATGCTCCACTTTCCTTGCATCAAATTTCGG<br>GCCAAGTAATTATTCAAACCCAACGAA	-	-	-	-	-	-	FAM	-	-
WriPdS25-1	GAAGGTGACCAAGTTCATGCTTACGATTGAAGCGTTTTTAAGGATYTCTGTAAC<br>GAAGGTCGGAGTCAACGGATTTACGATTGAAGCGTTTTTAAGGATYTCTGTAAT<br>CTTAACCAAATGCAATACTTCGAGCGATC	HEX	-	-	-	-	-	HEX	FAM	-
WriPdS25-2	GAAGGTGACCAAGTTCATGCTATGCTTAACCAAATGCAATACTTCGAGCGATCT<br>CTTTAATGGGTGATACTATGTCCGAGTACTTCCATA	-	-	-	-	-	-	-	FAM	-

[a] Allele-specific primers include tails (underlined) that are complementary to FRET cassettes in the KASP™ Master mix. Nucleotides for which the HEX or FAM tails overlap with allele-specific sequences are shown in bold font.

Table S11

Results of one-way multivariate analysis of variance analysis (MANOVA) for the KASP assay results shown in the left-hand panels of Supplementary Fig. S7. Clusters (genotype calls) were defined based on normalised FAM and HEX intensities for each of 15 KASP assays applied to a panel of almond cultivars. For each assay, *p* values are reported for both the Wald-type statistic (WTS) and the asymptotic model-based 'parametric' statistic (PBS). In cases for which two clusters were defined (degrees of freedom = 2) significance of WTS and PBS indicates significant separation between the two clusters. In cases for which three or four clusters were defined (degrees of freedom = 4 or 6), *p* values are also shown for *post hoc* Tukey contrasts for each pairwise comparison between clusters.

	**MANOVA**			***Post hoc* pairwise Tukey contrasts**					
		***p*-values**		***p*-values**					
**Marker**	**Degrees of freedom**	**WTS**	**PBS**	**HEX vs. FAM**	**HEX vs. HEX:FAM**	**HEX:FAM vs. FAM**	**HEX vs. Null**	**HEX:FAM vs. Null**	**FAM vs. Null**
WriPdSf-1	2	<0.001	<0.001						
WriPdSf-2	6	<0.001	<0.001	0.100	0.010	0.100	0.001	<0.001	<0.001
WriPdSf-3	4	<0.001	<0.001	<0.001	<0.001	<0.001			
WriPdSf-4	4	<0.001	<0.001	<0.001	<0.001	0.066			
WriPdSf-5	4	<0.001	<0.001	<0.001	<0.001	<0.001			
WriPdS1	2	<0.001	<0.001						
WriPdS3	2	<0.001	<0.001						
WriPdS5	2	<0.001	<0.001						
WriPdS7-1	2	<0.001	<0.001						
WriPdS7-2	2	<0.001	<0.001						
WriPdS8	2	<0.001	<0.001						
WriPdS9	2	<0.001	<0.001						
WriPdS23	2	<0.001	<0.001						
WriPdS25-1	2	<0.001	<0.001						
WriPdS25-2	2	<0.001	<0.001						

Table S12

Results of one-way multivariate analysis of variance analysis (MANOVA) for the KASP assay results shown in the right-hand panels of Supplementary Fig. S7. Clusters (genotype calls) were defined based on normalised FAM and HEX intensities for each of 15 KASP assays applied to F_1 progeny of relevant almond crosses. For each assay, *p* values are reported for both the Wald-type statistic (WTS) and the asymptotic model-based ‘parametric’ statistic (PBS). In cases for which two clusters were defined (degrees of freedom = 2), significance of WTS and PBS indicates significant separation between the two clusters. In cases where three or four clusters were defined (degrees of freedom = 4 or 6), *p* values are shown for *post hoc* Tukey contrasts for each pairwise comparison between clusters.

		**MANOVA**			***Post hoc* pairwise Tukey contrasts**					
		**Degrees of freedom**	***p*-values**		***p*-values**					
**Marker**	**Population**		**WTS**	**PBS**	**HEX vs. FAM**	**HEX vs. HEX:FAM**	**HEX:FAM vs. FAM**	**HEX vs. Null**	**HEX:FAM vs. Null**	**FAM vs. Null**
WriPdSf-1	Johnston’s Prolific ($S_{23}S_{25}$) × Lauranne (S_3S_f)	2	<0.001	<0.001						
WriPdSf-2	Maxima (S_3S_8) × Mira (S_7S_f)	6	<0.001	<0.001	<0.001	<0.001	0.078	0.001	<0.001	<0.001
WriPdSf-3	Nonpareil (S_7S_8) × Lauranne (S_3S_f)	4	<0.001	<0.001	<0.001	<0.001	0.047			
WriPdSf-4	Nonpareil (S_7S_8) × Lauranne (S_3S_f)	4	<0.001	<0.001	<0.001	<0.001	0.031			
WriPdSf-5	Maxima (S_3S_8) ×Vairo (S_9S_f)	4	<0.001	<0.001	<0.001	<0.001	<0.001			
WriPdS1	Carmel (S_5S_8) × 12-350 (S_1S_f)	2	<0.001	<0.001						
WriPdS3	Johnston’s Prolific ($S_{23}S_{25}$) × Lauranne (S_3S_f)	6	<0.001	<0.001	<0.001	<0.001	0.089	0.001	0.001	<0.001
WriPdS5	Carmel (S_5S_8) × Mandaline (S_1S_f)	2	<0.001	<0.001						
WriPdS7-1	Nonpareil (S_7S_8) × Mira (S_7S_f)	2	<0.001	<0.001						
WriPdS7-2	Nonpareil (S_7S_8) × Lauranne (S_3S_f)	2	<0.001	<0.001						
WriPdS8	Nonpareil (S_7S_8) × Lauranne (S_3S_f)	2	<0.001	<0.001						
WriPdS9	Nonpareil (S_7S_8) × Vairo (S_9S_f)	2	<0.001	<0.001						
WriPdS23	Johnston’s Prolific ($S_{23}S_{25}$) × 12-350 (S_1S_f)	2	<0.001	<0.001						
WriPdS25-1	Johnston’s Prolific ($S_{23}S_{25}$) × 12-350 (S_1S_f)	4	<0.001	<0.001	<0.001	<0.001	<0.001			
WriPdS25-2	Johnston’s Prolific ($S_{23}S_{25}$) × Vairo (S_9S_f)	2	<0.001	<0.001						

Table S13

Summary of results obtained from assessment of each of 15 KASP assays on F_1 progeny, showing the numbers of progeny for which HEX fluorescence, FAM fluorescence, both types of fluorescence (HEX:FAM) or no fluorescence (null) were detected

Marker	Population screened	Number of progeny			
		HEX	HEX:FAM	FAM	Null
WriPdSf-1	Johnston's Prolific ($S_{23}S_{25}$) × Lauranne (S_3S_f)	54	-	-	49
	Nonpareil (S_7S_8) × Lauranne (S_3S_f)	103	-	-	128
WriPdSf-2	Chellaston (S_7S_{23}) × Lauranne (S_3S_f)	94	58	44	-
	Johnston's Prolific ($S_{23}S_{25}$) × Lauranne (S_3S_f)	49	54	-	-
	Maxima (S_3S_8) × Mira (S_7S_f)	12	8	9	3
WriPdSf-3	Nonpareil (S_7S_8) × Lauranne (S_3S_f)	128	52	51	-
	Nonpareil (S_7S_8) × Marta (S_1S_f)	24	13	12	-
WriPdSf-4	Carmel (S_5S_8) × Capella (S_7S_f)	32	20	19	-
	Nonpareil (S_7S_8) × Lauranne (S_3S_f)	128	51	52	-
WriPdSf-5	Johnston's Prolific ($S_{23}S_{25}$) × Vairo (S_9S_f)	57	52	-	-
	Maxima (S_3S_8) × Vairo (S_9S_f)	75	28	41	-
WriPdS1	Carmel (S_5S_8) × 12-350 (S_1S_f)	-	-	40	52
	Antoñeta (S_1S_f) × Mira (S_7S_f)	-	-	86	97
WriPdS3	Johnston's Prolific ($S_{23}S_{25}$) × Lauranne (S_3S_f)	28	20	43	12
WriPdS5	Carmel (S_5S_8) × Francolí (S_1S_f)	-	-	96	102
	Carmel (S_5S_8) × Mandaline (S_1S_f)	-	-	31	36
WriPdS7-1	Nonpareil (S_7S_8) × Mira (S_7S_f)	23	24	-	-
	Maxima (S_3S_8) × Mira (S_7S_f)	16	16	-	-
WriPdS7-2	Nonpareil (S_7S_8) × Mira (S_7S_f)	-	-	24	23
	Nonpareil (S_7S_8) × Lauranne (S_3S_f)	-	-	129	102
WriPdS8	Nonpareil (S_7S_8) × Lauranne (S_3S_f)	-	-	129	102
	Carmel (S_5S_8) × Mandaline (S_1S_f)	-	-	36	31
WriPdS9	Nonpareil (S_7S_8) × Vairo (S_9S_f)	-	-	99	100
	Johnston's Prolific ($S_{23}S_{25}$) × Vairo (S_9S_f)	-	-	51	58
WriPdS23	Johnston's Prolific ($S_{23}S_{25}$) × 12-350 (S_1S_f)	-	-	61	66
	Johnston's Prolific ($S_{23}S_{25}$) × Vairo (S_9S_f)	-	-	54	55
WriPdS25-1	Johnston's Prolific ($S_{23}S_{25}$) × 12-350 (S_1S_f)	63	35	29	-
	Johnston's Prolific ($S_{23}S_{25}$) × Mandaline (S_1S_f)	4	4	4	-
WriPdS25-2	Johnston's Prolific ($S_{23}S_{25}$) × Vairo (S_9S_f)	-	-	47	62
	Johnston's Prolific ($S_{23}S_{25}$) × Constantí (S_3S_f)	-	-	35	34

Table S14

Ranges and means for the fruit set on bagged branches of trees for which self- fertility was predicted based on KASP marker results

Population	Marker(s) used to distinguish between self-incompatible and self-fertile trees	Trees predicted to be self-incompatible			Trees predicted to be self-fertile		
		Number of trees on which a branch was bagged[a]	Numbers of fruits on bagged branches		Number of trees on which a branch was bagged[a]	Numbers of fruits on bagged branches	
			Range	Mean		Range	Mean
Carmel (S_5S_8) × Capella (S_7S_f)	WriPdSf-4	8	0-2	0.6	20	8-65	23.1
Johnston's Prolific ($S_{23}S_{25}$) × Lauranne (S_3S_f)	WriPdSf-2	8	0-0	0	13	2-62	23.2
Maxima (S_3S_8) × Vairo (S_9S_f)	WriPdSf-5	10	0-0	0	19	8-52	27.7
Nonpareil (S_7S_8) × Lauranne (S_3S_f)	WriPdSf-3 and WriPdSf-4	8	0-0	0	20	13-72	25.4
Nonpareil (S_7S_8) × Marta (S_1S_f)	WriPdSf-3	8	0-2	0.4	20	8-67	23.0

[a] Excluding trees that failed to set any fruit on branches that were not bagged

Table S15
Evaluation of the protein models of five almond S-RNases and the MC1 RNase (PDB accession 1J1G) template

	Modeller		FoldX			ProSa 2003	PROCHECK		
			Total energy (kcal/mol)						
Model	DOPE	MOF	Before	After	Stability (kcal/mol)	Z-score	Allowed	Disallowed	G-factor
S_5-RNase	-22,152.12	3,100	45	38	39	-5.71	178	0	0.32
S_7-RNase	-23,150.71	3,200	42	37	39	-5.72	189	0	0.32
S_8-RNase	-22,870.35	3,800	41	37	38	-6.00	189	0	0.33
S_{23}-RNase	-23,867.35	3,756	41	38	39	-5.87	173	0	0.25
S_f-RNase	-22,858.25	3,100	41	33	37	-6.00	184	0	0.30
1J1G	-22,858.00	-	40	33	33	-6.72	162	0	0.35

Table S16

Secondary structure elements detected for five almond S-RNases and the MC1 RNase (PDB accession 1J1G)

Secondary structure elements		Protein					
		S_5-RNase	S_7-RNase	S_8-RNase	S_{23}-RNase	S_f-RNase	Bitter gourd seed RNase
α-helices	α1	PPTCAV	PTNCRV	PTTCRL	PTNCRV	PTTCRF	PTTCRF
	α2	-	-	-	-	-	GSC
	α3	TRFNN	KFDARK	QFNFMK	KFDARK	-	SPFDI
	α4	PKLEAKLKISW	PEMRSDLKISW	PQLRTKLKRSW	PKMRIKLKISW	RLRSKLERAW	KISHLQSQLNT
	α5	NYTEFWEREW	NDTKFWEDEW	NDTKFWEDEW	NDTRFWEDEW	NDTKFWEDEW	NQQFWSHEW
	α6	DQEEYFQRSHDIWNA	QFQYFERSHEMWMS	QMQYEEVSHAMWRS	QMQYFERSHQMWLS	QMQYFERSHQMWSS	QAAYFKLAVDMRNNY
	α7	ITNILKK	ITEILKN	ITNILKD	ITEILKN	ITNULEK	GALRP
	α8	YSDIVSPIKTVT	YSDIVSPIKAAT	YSDIVSPIKTAT	YSDIISPIKAAT	YSDILSPIKAAT	RQAIKGFLKAKF
β-sheets	β1	YFWFVQQ	YFWFVQQ	YFWFVQQ	YFWFVQQ	FVQQ	DFWFVQQ
	β2	FTIHG	FTIHG	FTIHG	FTIHG	TIH	FTIHGL
	β3	KAW	-	-	-	-	TSL
	β4	-	-	-	-	QTWT	TKS
	β5	LRCK	LLRCKY	LLRCK	LLRCK	LLRCK	GLR
	β6	LLHEVVLCH	LLHEVVLCG	LLHEVVLCG	LLHEVVLCE	FLHEVVLCY	YLVENN
	β7	LID	-	-	-	-	LID
	β8	KILF	KID	HIDILF	HIDISF	DIK	NFI
Loop anchoring points	α1	QWP	QWP	QWP	QWP	QWP	-
	α2	-	-	-	-	-	-
	α3	TRT	NGT	NGS	TGP	TGS	-
	α4	LA	VY	VY	VS	LS	-
	α5	ENA	ESG	EGG	ESG	ESG	-
	α6	QTL	QTL	RTL	ERL	QTL	-
	α7	YN	YN	YN	YN	FN	-
	α8	IWN	TWT	RWK	KWS	-	-
	β1	GSY	GSY	GSY	GSY	GSY	-
	β2	PSI	LQY	LQR	LQY	LPI	-
	β3	VAN	-	-	-	-	-
	β4	-	-	-	-	PNA	-
	β5	RTP	RTP	RTP	RTP	RIP	-
	β6	SHQ	NTQ	NSQ	NTL	TQF	-
	β7	GRA	-	-	-	-	-
	β8	NIK	CKN	CRN	GNK	NNV	-
Number of loops detected		12	11	11	11	11	-

Table S17

Sequences used to design primers in target regions of the almond *S* locus

Target region of the *S* locus (bp)	Sequences used for primer design																												
	**AB081587**	**AB101659_SLF**	**AB101660_SLF**	**AB433984_SRNase**	**AB011469_SRNase**	**AF490505_SRNase**	**DQ150569_SRNase**	**AM231657_SRNase**	**AY291118_SRNase**	**AB481108_SRNase**	**AF454001_SRNase**	**AM231662_SRNase**	**AM231663_SRNase**	**AM231668_Srnase**	**AM231671_SRNase**	**AB488496_SRNase**	**AM231673_SRNase**	**DQ099896_SFB**	**AB361036_SFB**	**AB092967_SFB**	**EU293149_SFB**	**FJ514936_SFB**	**FJ514937_SFB**	**FJ231204_SFB**	**FJ362529_SFB**	**AM746960_SFB**	**EU310403_SFB**	**FN429364_SFB**	**AB537564_SFB**	**Pp6 26-27Mb[a]**
307 bases upstream of the initial position of the AB081587 sequence − 10,010	X	X	X	X	X	X	X	X	X	X	X	X	X	X	X	X	X	X												
30 − 8,190	X	X	X	X	X	X	X	X	X	X	X	X	X	X	X	X	X	X	X	X	X	X	X	X	X	X	X	X		
7,750 − 12,460	X			X	X	X	X	X	X	X	X	X	X	X	X	X	X	X	X	X	X	X	X	X	X	X	X	X		
12,253 − 19,747	X																													X
19,154 − 32,182	X																												X	
31,890 − 71,953	X																													X

[a] The region from 26 Mb to 27 Mb of peach pseudomolecule 6

Table S18
Seven primer pairs designed to amplify regions of the almond *S* locus

**Primer pair**	**Target region of the *S* locus (bp)**	**Forward primer (5′-3′)**	**Reverse primer (5′-3′)**	**Annealing temperature (°C)**
WriPdSL7F/WriPdSL5R	68 bases upstream of the initial position of the AB081587 sequence − 8,156	CCAGAGYGACGCTAACAGTGATGAGA	CTTGGCCATADGCCATGGAT	63
WriPdSL9F/ WriPdSL13R	8,050 −16,448	CAATTTGTGCAACAATGGCC	ATGCCGACTAGTTCAATCAACA	63
WriPdSL13F/ WriPdSL15R	12,253 − 25,647	GGTATGCYRCTCCATTAAAA	TTCGTAGACACGGAGTAGACCC	65
WriPdSL16F/ WriPdSL17R	25,462 − 38,436	GTCGGGAAGGGAGGAAGGT	AAAAGAGAAGATCCGAGATGG	64
WriPdSL18F/ WriPdSL19R	38,211 − 50,573	AAACTGTAAGGACAACTTCGATTG	TGGACCTCCAGCAATAGTGA	65
WriPdSL20F/WriPdSL22R	50,258 − 63,144	TCACTGGACCTCCAGCAATA	AGGAAGGCGTATCGTTGAGA	65
WriPdSL24F/ WriPdSL25R	62,779 − 71,953	TGGAAGCACAGGTTTATGGA	CCTCAATAACTTTATCAGCTCC	64

Table S19
Approximate lengths of amplicons obtained with seven primer pairs, in addition to amplicons of the lengths expected for the S_7 haplotype

Primer pair	Cultivar or breeding selection (*S* genotype)									
	**Nonpareil (S_7S_8)**	**McKinlays (S_7S_8)**	**Capella (S_7S_f)**	**Mira (S_7S_f)**	**Carina (S_7S_f)**	**T5 (S_7S_f)**	**T7 (S_7S_f)**	**Brown Nonpareil (S_1S_7)**	**Baxendale (S_5S_7)**	**Keanes (S_7S_{27})**
WriPdSL7F/WriPdSL5R	8,300	8,400	8,400	8,400	8,400	8,400	8,400	8,400	5,300	3,400
WriPdSL9F/ WriPdSL13R	8,700	8,700	8,700	8,700	8,700	8,700	8,700	8,700	8,500	8,300
WriPdSL13F/ WriPdSL15R	12,000	12,000	12,000	12,000	12,000	12,000	12,000	12,000	12,000	12,000
WriPdSL16F/ WriPdSL17R	12,000	12,000	12,000	12,000	12,000	12,000	12,000	12,000	12,000	12,000
WriPdSL18F/ WriPdSL19R	10,000	10,000	10,000	10,000	10,000	10,000	10,000	12,000	10,000	10,000
WriPdSL20F/WriPdSL22R	12,000	12,000	12,000	12,000	12,000	12,000	12,000	12,000	12,000	12,000
WriPdSL24F/ WriPdSL25R	9,300	9,300	9,200	9,200	9,200	9,200	9,200	9,100	9,100	9,100

Table S20
Twenty-five primer pairs designed to amplify target regions of the almond *S* locus

Target region of the *S* locus (bp)	Primer pair	Forward primer (5′-3′)	Reverse primer (5′-3′)	Annealing temperature (°C)	Expected amplicon length (bp)
250 bases upstream of the initial position of the AB081587 sequence − 8,129	WriPdSL1	GTGGGAAGAGATGGCATTGCGC	TATTGTAATGGCCGGGGATTGGAGCA	64	8,379
307 bases upstream of the initial position of the AB081587 sequence − 8,129	WriPdSL2	AAACATATATTTCTAATCCCTCGTCG	TATTGTAATGGCCGGGGATTGGAGCA	64	8,436
307 bases upstream of the initial position of the AB081587 sequence − 8,129	WriPdSL3	AAASATATATTTCTAATCCCTCGTCG	TATTGTAATGGCCGGGGATTGGAG	65	8,436
210 bases upstream of the initial position of the AB081587 sequence − 10,033	WriPdSL4	GCCTTCAAAATCTCTAATGCGATTC	TTTGCAACGAAGGAGGGGTGT	65	10,243
210 bases upstream of the initial position of the AB081587 sequence − 8,156	WriPdSL5	GCSTTCAAAATCTCTAATGSGATTC	CTTGGCCATADGCCATGGAT	63	8,366
210 bases upstream of the initial position of the AB081587 sequence − 10,010	WriPdSL6	GCSTTCAAAATCTCTWATGSGATTC	TTCCAGTTGCTGCTTTRATG	65	10,220
68 bases upstream of the initial position of the AB081587 sequence − 8,072	WriPdSL7	CCAGAGYGACGCTAACAGTGATGAGA	GGTGGCCATTGTTGCACAAATT	65	8,197
30 − 8,190	WriPdSL8	GTTGAGGACATCAAGTTTCCACTTTC	GCAATTACTGSGCTTCSTTGGG	65	8,070
8,050 − 12,460	WriPdSL9	CAATTTGTGCAACAATGGCC	GGGGTTAATGACTACAAGRCTGT	63	4,410
7,914 − 12,293	WriPdSL10	CGCACAACTTTCTTTGGATR	ATACCACATCATTGAGAAAGGTC	63	4,381
7,913 − 12,293	WriPdSL11	ACGCACAACTTTCTTTGGATG	ATACCACATCATTGARAAAGGTC	63	4,380
7,750 − 12,383	WriPdSL12	GAGCASTGGTGGGTTRMATTAC	CAAWGATACCTTCGCGGTTG	63	4,470
12,253 − 16,448	WriPdSL13	GGTATGCYRCTCCATTAAAA	ATGCCGACTAGTTCAATCAACA	65	4,195
16,269 − 19,747	WriPdSL14	TWTGAGTGAATGACTTTTCTAAAGTTG	GGAAGATTTTGCGTTTGGTC	65	3,478
19,154 − 25,647	WriPdSL15	GTCCTGTGTCCGGCAATGAG	TTCGTAGACACGGAGTAGACCC	65	6,470
25,462 − 32,182	WriPdSL16	GTCGGGAAGGGAGGAAGGT	TTCATAAACGAGAAACCCATCA	65	6,700
31,890 − 38,436	WriPdSL17	CATTCATCGTGATCGGAATC	AAAAGAGAAGATCCGAGATGG	64	6,546
38,211 − 43,763	WriPdSL18	AAACTGTAAGGACAACTTCGATTG	GGTGTGGAGAGAAAGGTTGG	64	5,552
43,409 − 50,573	WriPdSL19	AGCGAACCTACTCCAACCATT	TGGACCTCCAGCAATAGTGA	65	7,164
50,258− 53,957	WriPdSL20	TCACTGGACCTCCAGCAATA	ATGGATGATTTGATTCGATTTT	65	3,699
53,638 − 59,851	WriPdSL21	CACCAAAGAAAATGGGTACGA	GCGAAAATAAAGTGGCATCG	65	6,213
51, 992 − 63,144	WriPdSL22	TCCTTCATGACTATTACATGTTCTCTT	AGGAAGGCGTATCGTTGAGA	65	11,152
62,786 − 68,214	WriPdSL23	ATTTGATGCAGCATGGAAGC	ATGTGAGAGATTTTAGCCTTTCATC	63	5,428
62,799 − 68,213	WriPdSL24	TGGAAGCACAGGTTTATGGA	TGTGAGAGATTTTAGCCTTTCATC	64	5,434
67,454 − 71,953	WriPdSL25	GCAGCAGTTTGTCATGAACC	CCTCAATAACTTTATCAGCTCC	63	4,499

Table S21
Amplicons obtained from 15 almond *S* haplotypes using 25 primer pairs

Primer pair	*S* haplotype[a]														
	S_1	S_3	S_5	S_6	S_7	S_8	S_9	S_{13}	S_{14}	S_{19}	S_{22}	S_{23}	S_{25}	S_{27}	S_f
WriPdSL1	■	□	■	□	□	□	□	□	□	□	□	□	□	□	□
WriPdSL2	□	■	□	□	□	□	□	□	□	□	□	□	□	□	□
WriPdSL3	□	□	■	□	□	□	□	□	□	■	□	■	□	□	□
WriPdSL4	□	■	□	■	□	□	□	□	□	□	□	□	□	□	□
WriPdSL5	□	□	■	□	■	■	■	■	■	■	■	■	■	■	■
WriPdSL6	□	□	■	□	□	□	□	□	■	■	□	□	□	■	□
WriPdSL7	■	■	■	□	■	■	□	■	■	■	□	■	■	■	■
WriPdSL8	□	□	■	■	■	□	□	■	■	■	□	■	■	■	□
WriPdSL9	■	■	■	□	□	□	□	□		□	□	□	□	□	-
WriPdSL10	■	-	■	■	■	■	-	■	□	■	■	■	■	■	■
WriPdSL11	■	-	■	-	■	■	-	-	-	■	■	■	■	-	-
WriPdSL12	■	■	■	■	■	■	■	■	□	■	■	■	■	■	■
WriPdSL13	■	■	■	■	■	■	■	■	■	■	□	■	■	■	■
WriPdSL14	■	■	■	■	■	■	■	■	■	□	■	■	□	■	■
WriPdSL15	■	■	□	■	■	■	■	-	-	-	■	■	■	■	■
WriPdSL16	■	■	■	■	■	■	■	-	□	□	□	□	□	■	■
WriPdSL17	■	-	-	-	■	■	■	■	-	-	-	■	■	-	■
WriPdSL18	■	■	■	-	■	■	■	■	-	-	-	■	-	-	□
WriPdSL19	■	■	■	-	■	■	■	■	-	-	-	■	-	-	□
WriPdSL20	■	■	■	-	■	■	■	■	-	■	-	■	■	□	■
WriPdSL21	■	-	-	□	■	■	■	-	□	■	-	■	■	-	□
WriPdSL22	■	■	■	-	■	■	■	□	-	■	-	■	■	□	■
WriPdSL23	■	-	■	-	■	■	■	-	-	-	□	■	■	-	□
WriPdSL24	■	■	■	-	■	■	■	□	-	□	-	■	■	□	■
WriPdSL25	■	■	■	■	■	■	■	■	■	■	■	■	■	■	■

[a]Boxes indicate the combinations of primer pairs and haplotypes for which an amplicon was obtained, while dashes indicate combinations for which no amplicon was obtained. Black boxes indicate that an amplicon was expected while white boxes indicate that no amplicon was expected.

Table S22
Almond populations used for the evaluation of *S*-locus marker assays

**Female parent (and *S* genotype)**	**Male parent (and *S* genotype)**	**Number of F_1 progeny**
Maxima (S_3S_8)	12-350 (S_1S_f)	232
Maxima (S_3S_8)	Vairo (S_9S_f)	144
Maxima (S_3S_8)	Mira (S_7S_f)	32
Antoñeta (S_1S_f)	Nonpareil (S_7S_8)	183
Antoñeta (S_1S_f)	Mira (S_7S_f)	183
Carmel (S_5S_8)	Francolí (S_1S_f)	198
Carmel (S_5S_8)	Mandaline (S_1S_f)	67
Carmel (S_5S_8)	Capella (S_7S_f)	71
Carmel (S_5S_8)	Vairo (S_9S_f)	100
Carmel (S_5S_8)	Antoñeta (S_1S_f)	76
Carmel (S_5S_8)	12-350 (S_1S_f)	92
Chellaston ((S_7S_{23})	Lauranne (S_3S_f)	196
Johnston's Prolific ($S_{23}S_{25}$)	12-350 (S_1S_f)	127
Johnston's Prolific ($S_{23}S_{25}$)	Mandaline (S_1S_f)	12
Johnston's Prolific ($S_{23}S_{25}$)	Lauranne (S_3S_f)	103
Johnston's Prolific ($S_{23}S_{25}$)	Vairo (S_9S_f)	109
Johnston's Prolific ($S_{23}S_{25}$)	Capella (S_7S_f)	185
Johnston's Prolific ($S_{23}S_{25}$)	Constantí (S_3S_f)	69
Nonpareil (S_7S_8)	Lauranne (S_3S_f)	231
Nonpareil (S_7S_8)	Marta (S_1S_f)	49
Nonpareil (S_7S_8)	Vairo (S_9S_f)	199
Nonpareil (S_7S_8)	12-350 (S_1S_f)	135
Nonpareil (S_7S_8)	Constantí (S_3S_f)	350
Nonpareil (S_7S_8)	Mira (S_7S_f)	47
Somerton (S_1S_{23})	Capella (S_7S_f)	94
Somerton (S_1S_{23})	Mira (S_7S_f)	133

Figure S1

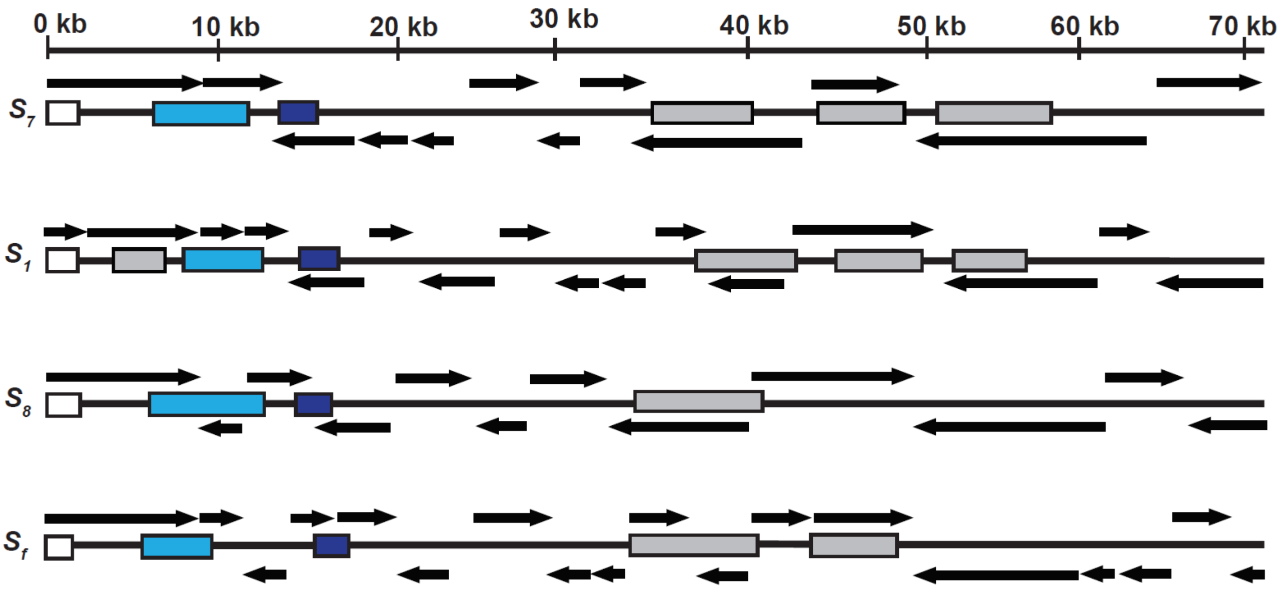

Open reading frames (ORFs) detected for the S_7-, S_1-, S_8- and S_f-haplotype sequences. For each haplotype, the *SLF* gene (white) *S-RNase* gene (cyan), *SFB* gene (dark blue) and long-terminal-repeat retrotransposons (grey) are shown, along with the ORFs (arrows). The strands on which those ORFs were detected are indicated by forward and reverse arrows.

Figure S2

```
SLF1    1 DAEHNVNFVVEDIKFPLSSGRFIGLEDVESPSILGHCNGIVCLSPCSDNLVLCNPSIKEIKLLPKSGLPDWWGCALGFGYDPKSKNYKVSRIASYQAEIDGLIPPPRVEIYTLSTDSWRE 120
SLF3    1 DAEHNVNFIVEDIKFPLSSGRFIGLEDVESPSILGHCNGIVCLSPCSDNLVLCNPSIKEIKLLPKSGLPDWWGCAVGFGYDPKSKNYKVSRIASYQAEIDGLIPPPRVEIYTLSIDSWRE 120
SLF5    1 DAEHNVNFVVEDIKFPLSSGRFIGLEDVESPSILGHCNGIVCLSPCSDNLVLCNPSIKEIKLLPKSGLPDWWGCAVGFGYDPKSKNYKVSRIASYQAEIDGLIPPPRVEIYTLSIDSWRE 120
SLF6    1 DAEHNVNFVVEDIKFPLSSGRFIGLEDVESPSILGHCNGIVCLSPCSDNLVLCNPSIKEIKLLPKSGLPDWWGCAVGFGYDPKSKNYKVSRIASYQAEIDGLIPPPRVEIYTLSTDSWGE 120
SLF7    1 DDEHNLNFVVEDIKFPLSSGQFIGLEDVESPSILGHCNGIVCLSPCSDNLVLCNPAIKEIKLLPKSGLPDWWGCAVGFGYDPKSKDYKVSRIASYQVEIDGLIPPPRVEIYSLSTDSWRE 120
SLF8    1 DDEHNVNFVVEGNGFSLSSGRFIGLEDVESPSILGHCNGIVCLSPCSDNLVLCNPAIKEIKLLPKSGLPDWWGCAVGFGYDPKSKDYKVSRIASYQAEIDGLIPPPRVEIYTLSTDSWRE 120
SLF9    1 DDEHNVNFVVEDIKFPLSSGQFIGLEDVESPSILGHCNGIVCLSPCSDNLVLCNPSIKEIKLLPKSGLPDWWGCAVGFGYDPKSKNYKVSRIASYQAEIDGLIPPPRVEIYTLSIDSWRE 120
SLF13   1 DAEHNVNFVVEDIKFPLSSGRFIGLEDVESPSILGHCNGIVCLSPCSDNLVLCNPSIKEIKLLPKSGLPDWWGCAVGFGYDPKSKNYKVSRIASYQAEIDGLIPPPRVEIYTLSTDSWGE 120
SLF14   1 DAENNVNFVVEDIKFPLSSGQFIGLDVIESPSIIGHCDGVICLSACSGNLVLCNPAINEIKLLPGSCLPDWWDCAVGFGYDPKSKDYKVNRIATYQAEIDGLIPPPRVEIYSLSTDSWRE 120
SLF19   1 DAEHNVNFVVEDIKFPLSSGRFIGLEDVESPSILGHCNGIVCLSPCSDNLVLCNPAIKEIKLLPKSGLPDWWGCAVGFGYDPKSKNYKVSRIASYQAEIDGLIPPPRVEIYTLSIDSWRE 120
SLF22   1 DDEHNLNFVVEDIKFPLSSGRFIGLEDVESPSILGHCNGIVCLSRCSDNLVLCNPSIKEIKLLPKSGLPDWWGCAVGFGYDLKSKNYKVSRIASYQAEIDGLIPPPRVEIYTLSTDSWGE 120
SLF23   1 DAEHNVNFVVEDIKFPLSTGLFIGLEDVESLSILGHCNGIVCLSRCSDNHVLCNPSIKELKLLPKSCLPDWWGCAVGFGYDPKSKNYKVSRIASYQAEIDGLIPPPRVEIYTLCIDSWRE 120
SLF25   1 DDEHNVNFIVEDIKFPLSSGRFIGLEDVESPSILGHCNGIVCLSPCSDNLVLCNPAIKEIKLLPKSGLPDWWGCALGFGYDPKSKAYKVSRIASYQAEIDGLIPPPRVEIYTLSTDSWRE 120
SLF27   1 DAEHNVNFVVEDIKFPLSSGRFIGLEDVESPSILGHCNGIVCLSPCSDNLVLCNPAIKEIKLLPKSGLPDWWGCAVGFGYDPKSKNYKVSRIASYQAEIDGLIPPPRVEIYTLSIDSWRE 120
SLFf    1 DAEHNVNFIVEDIKFPLSSGRFIGLEDVESLSIIGHCDGIICLSTCSGNIVLCNPAINELKLLPKSCLTDWWGCALGFGYDLKSKDYKVSRIATYQVEIGGLIPPPRVEIYNLRTDSWRE 120

SLF1  121 IKNNCLETDTTCFFPDYFQMYFQGICYWVGYDQPKQSVEYEDEEQKPMVILFDTGDEIFHNILLPDSFYMYEEGSAYAYEMSYIMYSDLRIILWNGSVALFGFNPCSALPESYGVWVLDG 240
SLF3  121 IKNNCLETDTTCFFPDYFQMYFQGICYWVGYDQPKQSVEYEDEEQKPMVILFDTGDEIFHNLLLPDSFYMYEEGSSYAYEMSYLMYSDLRIILWNGSITLFGFNPCSVFPDSYGVWVLDG 240
SLF5  121 IKNNCLETDTTCFFPDYFQMYFQGICYWVGYDQPKQSVEYEDEEQKPMVILFDTGDEIFNNILLPDSFYMYEEGSAYAYEMSYIMYSDLRIILWNGSVALFGFNPCSALPESYGVWVLDD 240
SLF6  121 IKNNSLETDTTCFFPDYFQMYFQGICYWVGYEQPKQSVEYEDEEQKPMVILFDTGDEIFHNILLPDSFYMYEEGSAYAYEMSYIMYSDLRIILWNGSVALFGFNRFSAFPDSYGVWVLDG 240
SLF7  121 IKNNSLETDTTCFFPDYFQMYFQGICYWVGYEQPKQSVEYEDEEQKPMVIFFDTGDEIFNHILFPDSFYMYEEGSSYAYEMSYLMYCDLRIILWNGSIALFGFNRFSVFPDSYGVWVLDD 240
SLF8  121 INNNSLETDSTCFFPDYFQMYFQGICYWVGYEQPKQSVEYEDEEQKPMVIFFDTGDEIFHNLLFPDSFYMYEEGSSYAYEMSYLMYCDLRIILWNGSIALFGFNRFSVFPDSYGVWVLDD 240
SLF9  121 IKNNCLETDTTCFFPDYFQMYFHGICYWVGYDQPKQSVEYEDEEQKPMVILFDTGDEIFHHILLPDSFYMYEEGSSYAYEMSYLMYSDLRIILWNGSITLFGFNPCSVFPDSYGVWVLDG 240
SLF13 121 IKNNSLETDTTCFFPDYFQMYFQGICYWVGYEQPKQSVEYEDEEQKPMVILFDTGDEIFHNILLPDSFYMYEEGSAYAYEMSYIMYSDLRIILWNGSVALFGFNRFSAFPDSYGVWVMDG 240
SLF14 121 IKNNSLETDTTCFFPDYFQMYFKGICYWVGYEQPKQSVEYEDEEQKPMVIFLDTGDEIFHHILFPDSFYMYEEGSSYAYEMSYIMYTDLRIILWNGSIALFGFNRFSAFPDSYGVWVLDG 240
SLF19 121 IKNNCLETDTTCFFPDYFQMYFHGICYWVGYDQPKQSVEYEDEEQKPMVILFDTGDEIFHNILLPDSFYMYEEGSAYAYEMSYIMYSDLRIILWNGSIALFGFNPCSAFPESYGVWVLDD 240
SLF22 121 IRNNSLETGTTCFFPDYFQMYFQGICYWVGYEQPKQSVEYEDEEQKPMVIFFDTGDEIFHHILFPDSFYMYEEGSSYACEMSYIMYTDLRIIPWNGFVALFGINRFSALPDSYGVWVLDG 240
SLF23 121 MRNNWLETDTTCFFPDYFQMYFHGICYWVGYDQPKQSVEYEDDEKKPMVILFDTGDEIFHNILLPDSFYMYEEGSTFAYEMSYIMCSDMRIIPWNGFVALFGINPCSALPDSYEVWVLDG 240
SLF25 121 IKNNSLETDTTCFFPDYFQMYFQGICYWVGYEQPKQSVEYEDEEQKPMVLFFDMSDEIFHNILFPNSFYMYEEGSAYAYEMSYLMYCDLRIILWNGSIALFGFSRFSVFPESYGVWVLDD 240
SLF27 121 IKNNCLETDTTCFFPDYFQMYFHGICYWVGYDQPKQSVEYEDEEQKPMVILFDTGDEIFHNILLPDSFYMYEEGSAYAYEMSYIMYSDLRIILWNGSIALFGFNPCSAFPESYGVWVLDD 240
SLFf  121 IKNNSLETGTTCFFPDYFQMYFQGICYWVGYEQPKQSVEYEDEEQKPMVIFFDTGDEKFHNILFPDSFYMYEEGSAYAYEMSYIMYCDLRIILWNGSVALFGINRFSALPESYGVWVLDD 240

SLF1  241 FDGAKGSWTKHLTFEPLIGIKRVLEFWKSDEILMVTENGHVVSYNLETEKLKNLPMNNPSDFETIVYVNSLVPITGGNKLENVDI 325
SLF3  241 FDGAKGSWTKHLTFEPLMGIKRVLEFWRSDEILMVTEDGDVVSYNLETEKLKNLPMNSLSDFETIVYVNSLVSITRGNKLESVDI 325
SLF5  241 FDGAKGSWTKHLTFEPLFGIKRVLEFWKSDEILMVTEHGHVVSYNLETEKLKNLPMNIPSDFETIVYVNSLVLITGGNKLENVDI 325
SLF6  241 FDGAKGSWTKHLTFEPLFGIKRVLEFWKSDEILMVTEDGDIVSYNLATEKLKNLPMNSLSDFETIVYVNSLVPITGGNKLENVDI 325
SLF7  241 FDGAKGSWTKHLTFEPLMGIKRVLEFWRSDEILMVTEDGDIVSYNLATQKLENLPMNSLSDFETIVYVNSLVSITRGNKLESVDI 325
SLF8  241 FDGAKGSWTKHLTFEPLMGIKRVLEFWRSDEILMVTEDGDIVSYNLATQKLENLPMNSLSDFETIVYVNSLVSITRGNKLESVDI 325
SLF9  241 FDGAKGSWTKHLTFEPLIGIKRVLEFWRSDEILMVTENGDVVSYNLETEKLKNLPMNSPSDFETIVYVNSLVSITRGNKLESVDI 325
SLF13 241 FDGAKGSWTKHLTFEPLFGIKRVLEFWKSDEILMVTEDGHVVSYNLATEKLKNLPMNSPSDFETIVYVNSLVLITGGNKLENVDI 325
SLF14 241 FDGAKASWTKHLTVDPLVGIKRVLEFWKGDEILMVTENGHIVAYNLETEKLKNLTMNISSDFETIVYVNSLVPITGGNKIESVDI 325
SLF19 241 FDGAKGSWTKHLTFEPLFGIKRVLEFWTSDEILMVTENGHVVSYNLETEKLKNLPMNSPSDFETIVYVNSLVLITGGNKLENVDI 325
SLF22 241 FDGAKGSWTKHLTFDPLVCIKRVLEFWKSDEILMVTEDGDIVSYNLATEKLKNFPMNSSSDFETNVYVNSLVWITRDIKLEAVDI 325
SLF23 241 FDGAKGSWTKHLTFDPVIDIKRVLEFWTTDEILMVTENGHVVTYNLETEKLKNLHMNILSDFETIVYVNSLVLITGGIKLETVDI 325
SLF25 241 FDVAKRSWTKHLGFEPLVGIERVLEFWRSDEILMATEDEDIVSYNLATEKLKNLPMNSPSDFETIVYVNSLVSITRGNKLENVHI 325
SLF27 241 FDGAKGSWTKHLTFEPLFGIKRVLEFWTSDEILMVTENGHVVSYNLETEKLKNLPMNSPSDFETIVYVNSLVLITGGNKLENVDI 325
SLFf  241 FDGAKVSWIKHLVFEPLVGIKRVLEFWKSDEILIATEDGYIVSYNLATEKLKNLPMNSLSDFETILYVNSLVWITGGNKLETVHI 325
```

Sequence alignment of 15 almond *S* locus F-box proteins. Absolutely conserved amino acid residues are indicated by black arrowheads above the alignment.

Figure S3

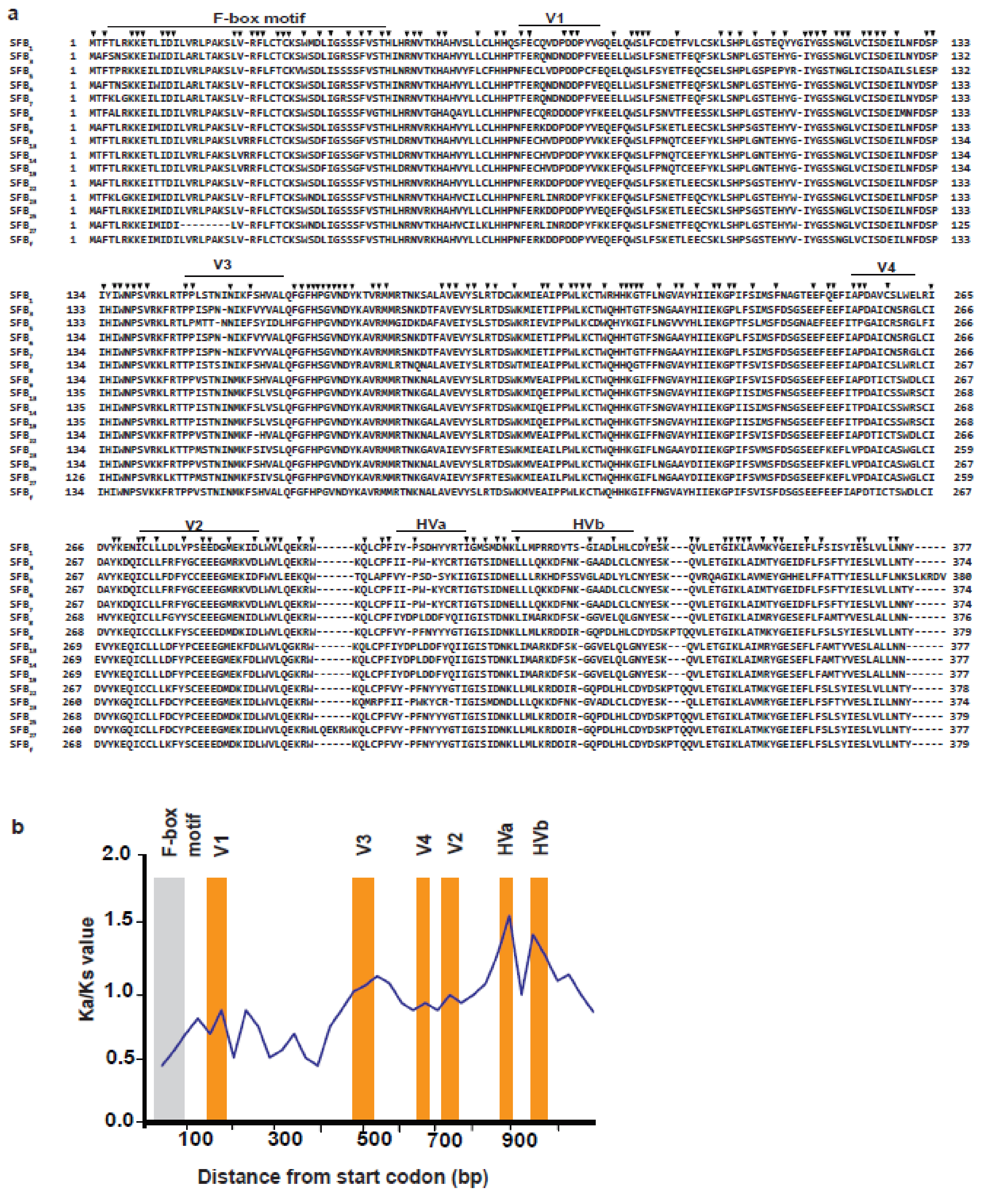

SFB protein sequence alignment and Ka/Ks ratios. (**a**) Sequence alignment of 15 almond SFB proteins, showing variable regions (V1-V4) and hypervariable regions (HVa and HVb) are shown. Positions of absolutely conserved residues are indicated by arrowheads above the alignment. (**b**) Mean nonsynonymous/synonymous (Ka/Ks) ratios for coding regions of the *SFB* gene of almond, calculated for 100 bp sliding windows with a 20 bp step size. The F-box motif is highlighted in grey and variable (V1–V4) or hypervariable regions (HVa and HVb) of the *SFB* gene are highlighted in orange.

Figure S4

```
        397                                                                                                                                                                                                 594
S1      AT--ATAAAATTTTG--------------------------------------GTGTTGGTTTAGTTTTTTTAATTATTATTTTTTTAATA-TTGGTTCTTAGTTAGACACATTATTTTGAAA-ATA   TAGTCAAGTTC-AAGATGG--TAC-GTAT---ATTTATATTTATATGCATATTAATGTACTTGCGAATATA
S3      AAGATCTATTATCTGATTTTTTTTTTTAAAAAAAAAATTTTTTAAATATGAAAAAGTGTGAATTTACCATATTATCCTCATTTAATTAATAATTTTAATTCTTATCGTTTACATTAACAAATGGCATTT   TTTGGTATTTTGAATGTTTCAC-TATTCTCTGCCTTTTGCTTTTTATATATATTGATTTAGTTTTTACATG
S5      -------------------------------------------------------------------------------------------------------------------------------   -----------------------------------------------------------------------
S7      C-GATATGTTTTCACGTTTGCTTCTCCTA------------------------GTGCTTCCCTAC--TTTC--GGCCCTCTCTCTACCGTCCCCTCTCCCTGGGCTCTCCACAGGCACTCCATGAAC   ACACTCCATTCTCCATATATCTTCCTCCGTTAAATAAACACCATCGTTCCTATTGTCTAAACTTTAATAGA
S8      TTTTTT-----------------------------------------------GTGACACTAATGATTTTTAAGGTTAC-TCTCTACACATCAAAATGGTCATTGCCATTAGCTTCCGTCAAATTTT   ATGTTAAATTGATAATGTGGCACATATGTGGAGCCTACACATACAATAGTATATAGTCACATGACTTCATG
S9      AGGCACGTTATTTTGAATATATATAA---------------------------GTACATTTATATTCGTT---GGTAT--ATTAC-G---TATATAATTTAGTTTTTTTTTTGGGGTCA-AAATTAA   -AAAGAAAAAGAAAATTTCTCACACACACACAATTAAGGTGTCATGGAGGTTCGAACCTGAGGCCATTAAG
S23     TA-------TTATTA--TTATTATTATTTTTTTGG------------------GTTGGTAACAAGAGGGGCTAAGAGCCCAGAAC-------AACAA-GACCCCAAACACATTATT-------TTGA   AT-AT-ATAGTTAAGTACAAAATGG---TAAAGTAT---ATTTATATTTATATG-----------CATAA
S25     CTCATATTTGGGAGCTCCTAGAGATCTTCTTATATTAGGAG------------GTAGATAGGGAGCATGATACTTGTATTTTTCCAATTTCTCCCTAAAATT----T-GTTTAAG----AAAG--TG   TTTTAGAGAAC-AATATTGCAGATGCTCTTAGTTTTATAAAATTTGATTGTCGTGAAGACAATATACATAC
Sf      TATATTAATGCAACTAGTCATGCATTTATTTCATG ------------------GTGCCCTATCTAATTTGTTGACATATGTC ATTCCACTAACTTTTAAACTTAACACTGGT AACTTTTAATACAATTAGAAAAACAAAAATTAATTATTAAAGAAATAACAAAAATCCAACAGCCATCACAACACCCCACCCACCCA
           ----------------------------->                                             <-----------------------------
                     WriPdSf-1                                                                    WriPdSf-1
```

Primer design for a marker assay to distinguish S_f from other almond *S-RNase* alleles. A 198 bp region of the *S-RNase* gene showing aligned sequences for the S_f self-fertility allele and eight SI alleles, with annealing sites for the WriPdSf-1 primers shown in white text on a black background.

Figure S5

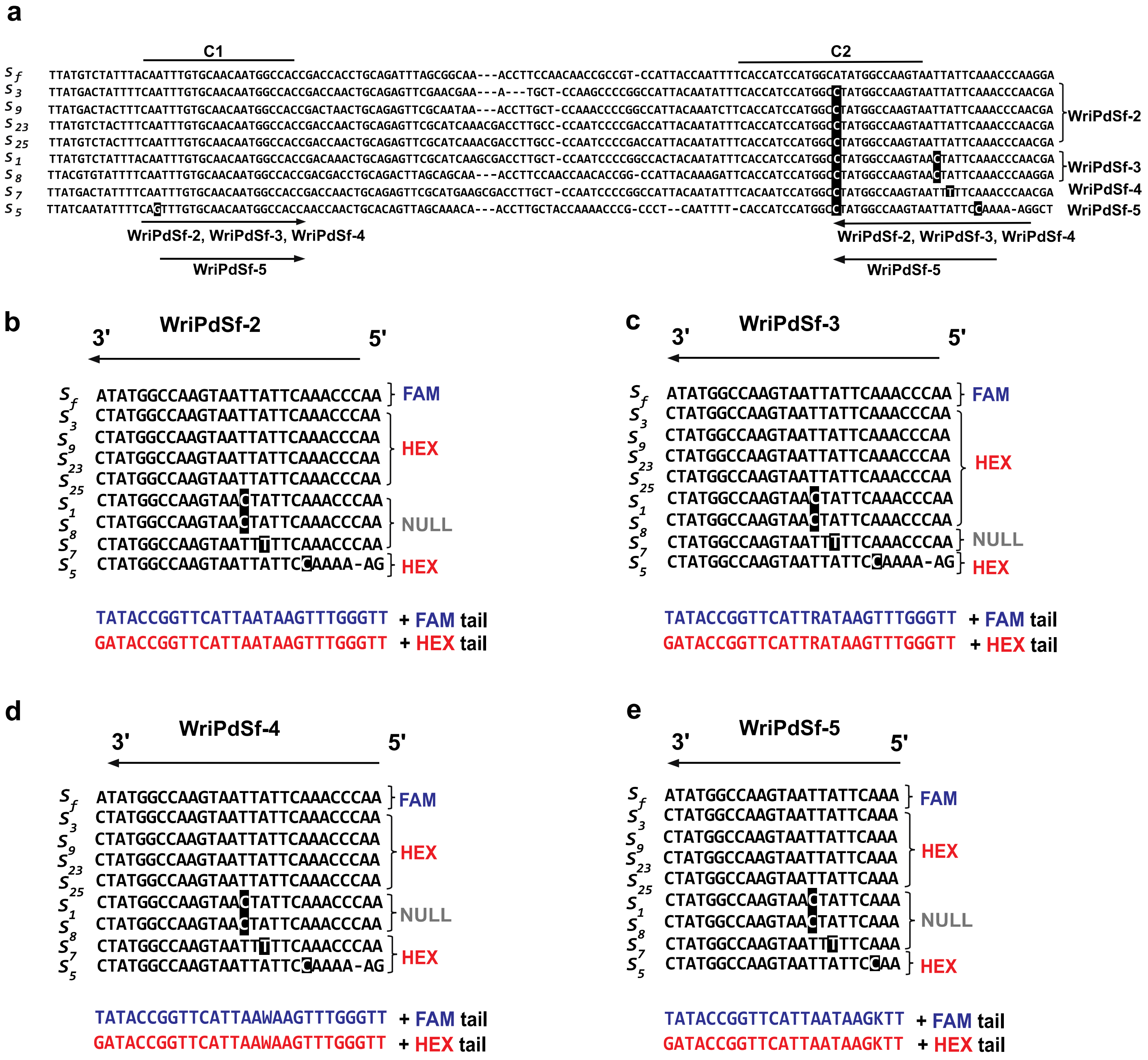

Primer design for four marker assays to distinguish S_f from other almond *S-RNase* alleles. (**a**) A 147 bp region of the *S-RNase* gene showing aligned sequences for the S_f self-fertility allele and eight SI alleles, showing the position of a single nucleotide polymorphism that distinguishes the S_f allele (A) from all eight SI alleles (C, shown in white text on a black background). Primer annealing sites for four marker assays (WriPdSf-2, WriPdSf-3, WriPdSf-4 and WriPdSf-5) are shown by arrows. (**b**) Sequences of the FAM-tailed and HEX-tailed allele-specific primers used in marker assay WriPdSf-2 and the expected fluorescence type (FAM, HEX or NULL) for each of nine *S-RNase* alleles. (**c**) Sequences of the FAM-tailed and HEX-tailed allele-specific primers used in marker assay WriPdSf-3 and the expected fluorescence type (FAM, HEX or NULL) for each of nine *S-RNase* alleles. (**d**) Sequences of the FAM-tailed and HEX-tailed allele-specific primers used in marker assay WriPdSf-4 and the expected fluorescence type (FAM, HEX or NULL) for each of nine *S-RNase* alleles. (**e**) Sequences of the FAM-tailed and HEX-tailed allele-specific primers used in marker assay WriPdSf-5 and the expected fluorescence type (FAM, HEX or NULL) for each of nine *S-RNase* alleles.

Figure S6

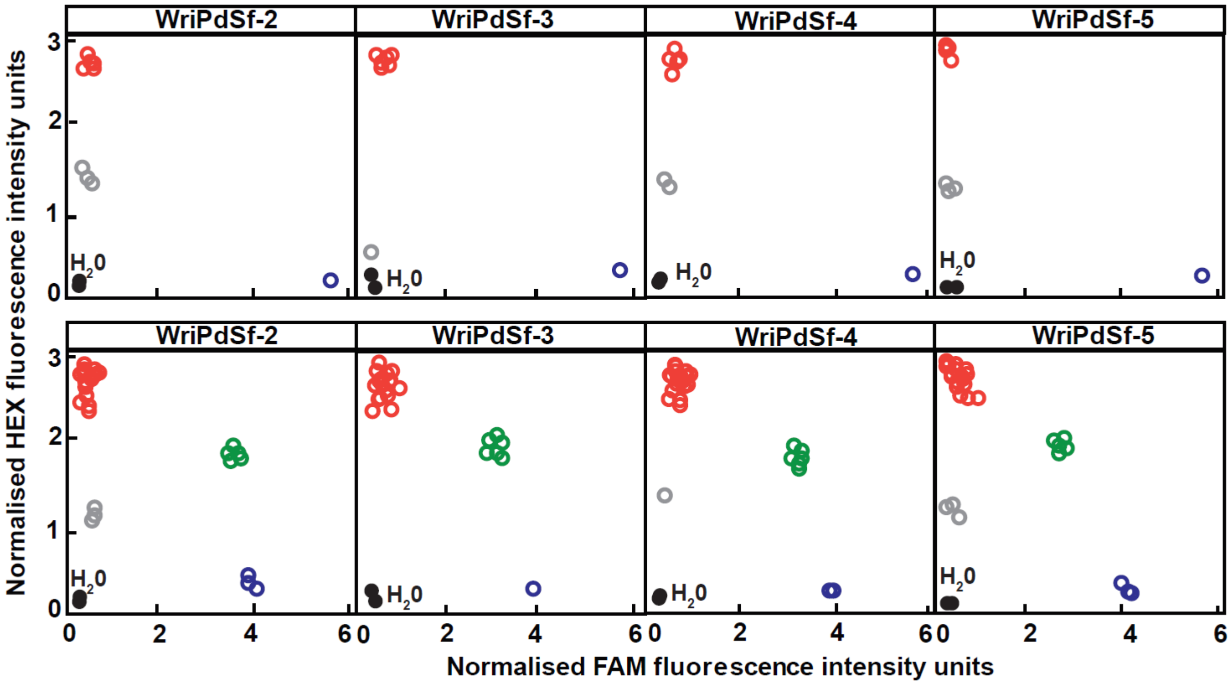

Results obtained with four primer sets applied to samples of synthesized DNA representing almond *S-RNase* alleles and 1:1 mixtures representing heterozygous genotypes. Data shown are intensities of FAM and HEX fluorescence, each normalised against fluorescence from an internal ROX reference. The results in the upper panel are for pure DNA samples representing nine almond *S-RNase* alleles (S_1, S_3, S_5, S_7, S_8, S_9, S_{23}, S_{25} and S_f). With each primer set, strong FAM fluorescence (blue data points) was detected for S_f, weak HEX fluorescence (grey data points) was detected for up to three SI alleles (S_1, S_7 and S_8 for WriPdSf-2 and WriPdSf-5; S_7 for WriPdSf-3; S_1 and S_8 for WriPdSf-4) (null alleles) and strong HEX fluorescence (red data points) was detected for all other SI alleles (HEX alleles). The results shown in the lower panel are for 1:1 mixtures representing the possible heterozygotes involving these alleles. Strong FAM fluorescence was detected for each mixture of S_f with a null allele. Both FAM and HEX fluorescence (green data points) were detected for each mixture of S_f with a HEX allele. Strong HEX fluorescence was detected for each mixture of two HEX alleles and for each mixture of a HEX allele with a null allele. Weak HEX fluorescence was detected for each mixture of two null alleles.

Figure S7

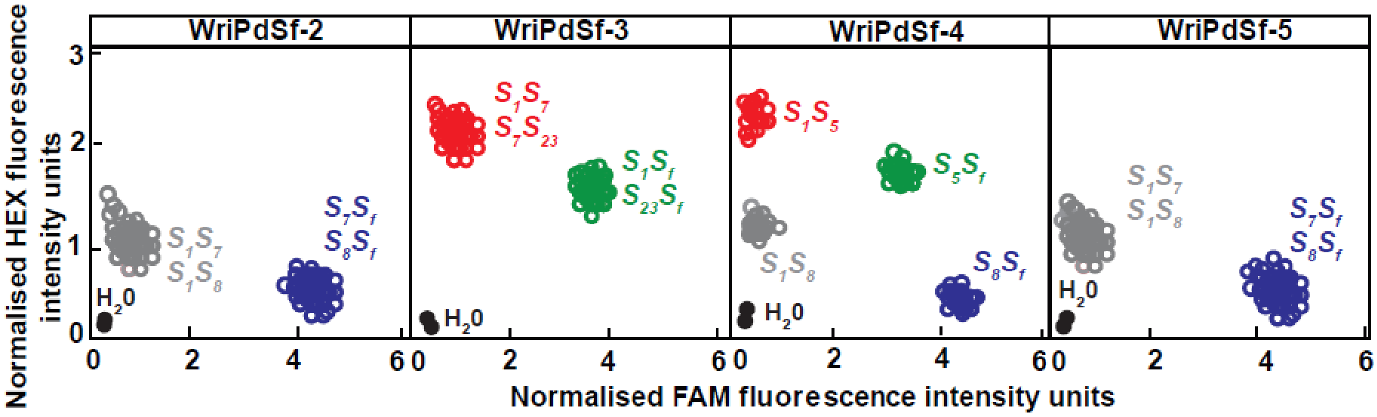

Results obtained with four primer sets, each applied to F_1 progeny from one cross. WriPdSf-2 and WriPdSf-5 applied to Antoñeta (S_1S_f) x Nonpareil (S_7S_8), WriPdSf-3 applied to Somerton (S_1S_{23}) x Mira (S_7S_f) and WriPdSf-4 applied to Carmel (S_5S_8) x Antoñeta (S_1S_f). Data shown are intensities of FAM and HEX fluorescence, each normalised against fluorescence from an internal ROX reference.

Figure S8

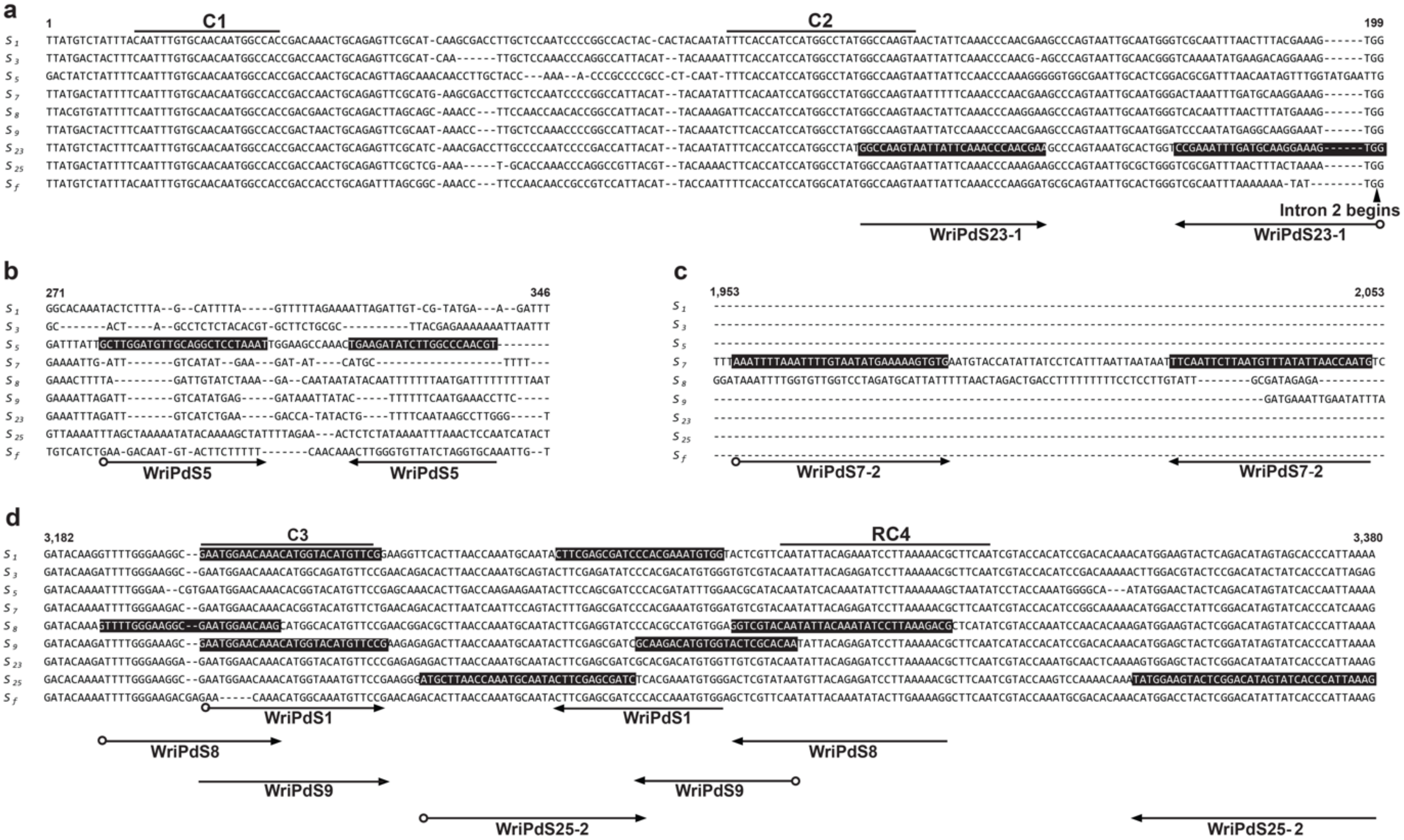

Primer design for two-primer assays to distinguish among *S-RNase* alleles. Aligned sequences of nine alleles in four regions (**a**, **b**, **c** and **d**) of the *S-RNase* gene, with positions of conserved regions (C1, C2, C3 and RC4) shown. Annealing sites for primers are shown in white text on a black background and with arrows, labelled with the names of the primer pairs to which they belong. Each primer pair consists of one untailed primer (simple arrow) and one primer to which a FAM tail was attached at the position shown by a circle.

Figure S9

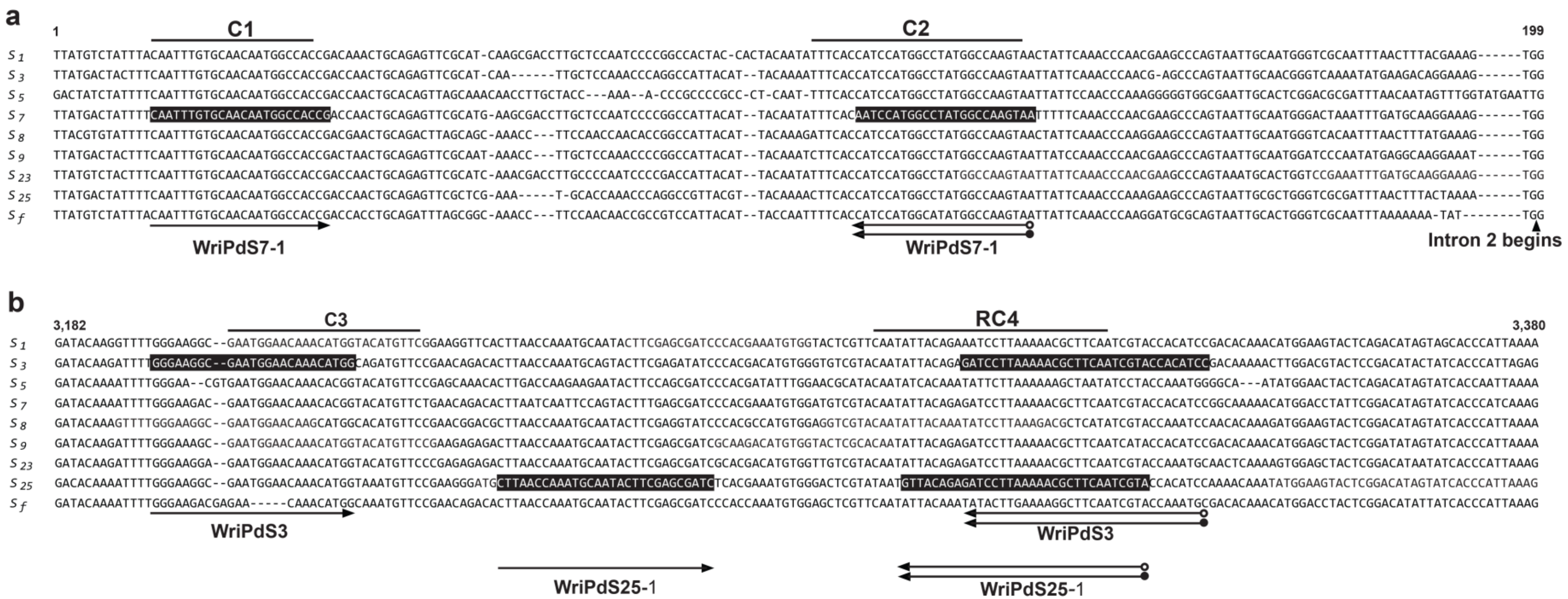

Primer design for three-primer assays to distinguish among *S-RNase* alleles. Aligned sequences of nine alleles in two regions (**a** and **b**) of the *S-RNase* gene, with positions of conserved regions (C1, C2, C3 and RC4) shown. Annealing sites for primers are shown in white text on a black background and with arrows, labelled with the names of the primer sets to which they belong. Single arrows indicate common untailed primers. Pairs of arrows indicate pairs of allele-specific primers. For each pair of allele specific primers, a FAM tail was added to one primer and a HEX tail was added to the other primer, at the positions shown by white and black circles, respectively.

Figure S10

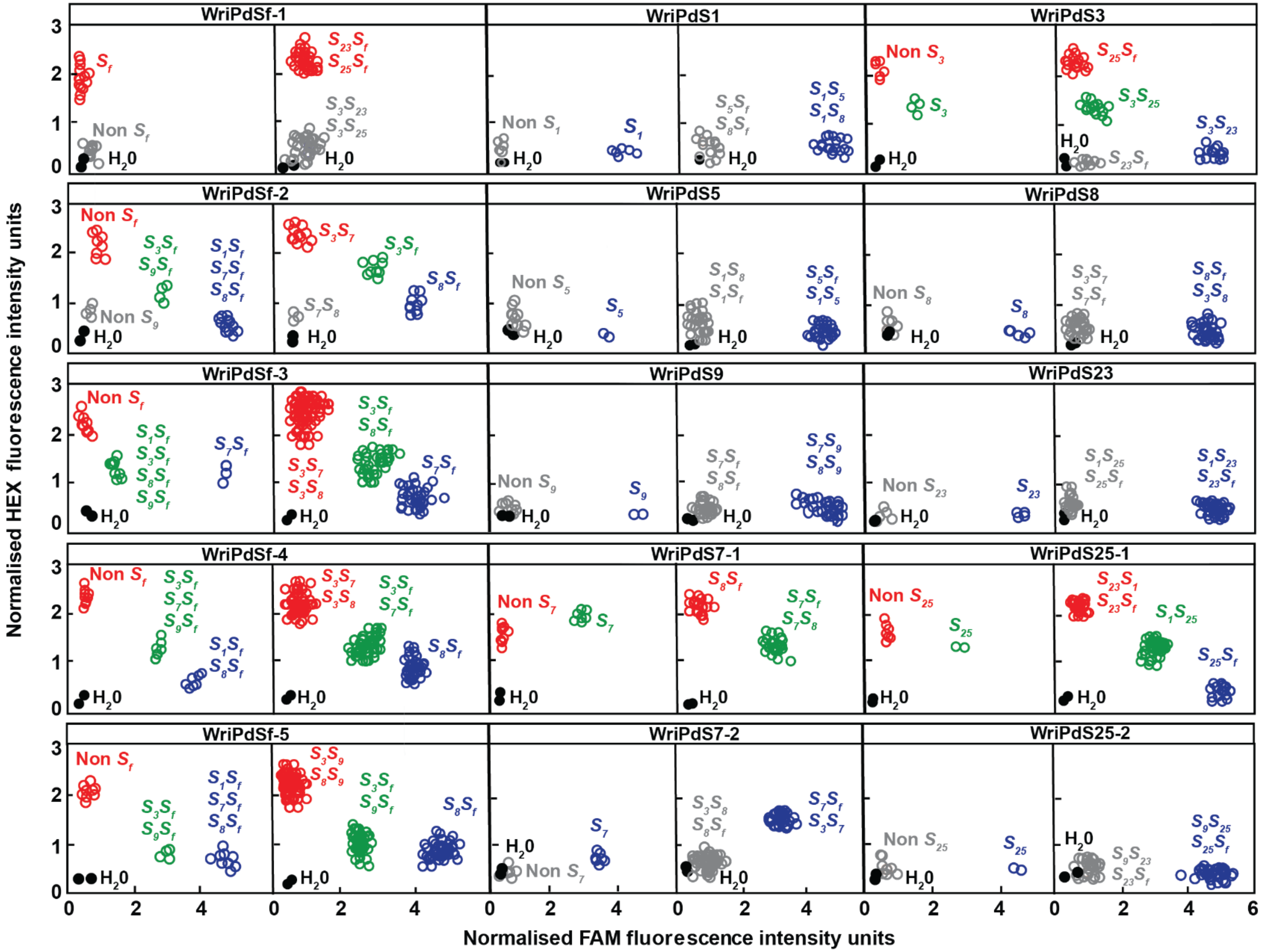

Results obtained with 15 primer sets designed to distinguish among *S-RNase* alleles of almond. For each primer set, the left-hand panel shows results obtained for a set of almond cultivars and the right-hand panel shows results obtained on F_1 progeny from a relevant cross:

- Johnston's Prolific ($S_{23}S_{25}$) × Lauranne (S_3S_f) for WriPdSf-1 and WriPdS3
- Maxima (S_3S_8) × Mira (S_7S_f) for WriPdSf-2
- Nonpareil (S_7S_8) × Lauranne (S_3S_f) for WriPdSf-3, WriPdSf-4, WriPdS7-2 and WriPdS8
- Maxima (S_3S_8) ×Vairo (S_9S_f) for WriPdSf-5
- Carmel (S_5S_8) × 12-350 (S_1S_f) for WriPdS1
- Carmel (S_5S_8) × Mandaline (S_1S_f) for WriPdS5
- Nonpareil (S_7S_8) × Mira (S_7S_f) for WriPdS7-1
- Nonpareil (S_7S_8) × Vairo (S_9S_f) for WriPdS9
- Johnston's Prolific ($S_{23}S_{25}$) × 12-350 (S_1S_f) for WriPdS23 and WriPdS25-1
- Johnston's Prolific ($S_{23}S_{25}$) × Vairo (S_9S_f) for WriPdS25-2

Data shown are intensities of FAM and HEX fluorescence, each normalised against fluorescence from an internal ROX reference.

Figure S11

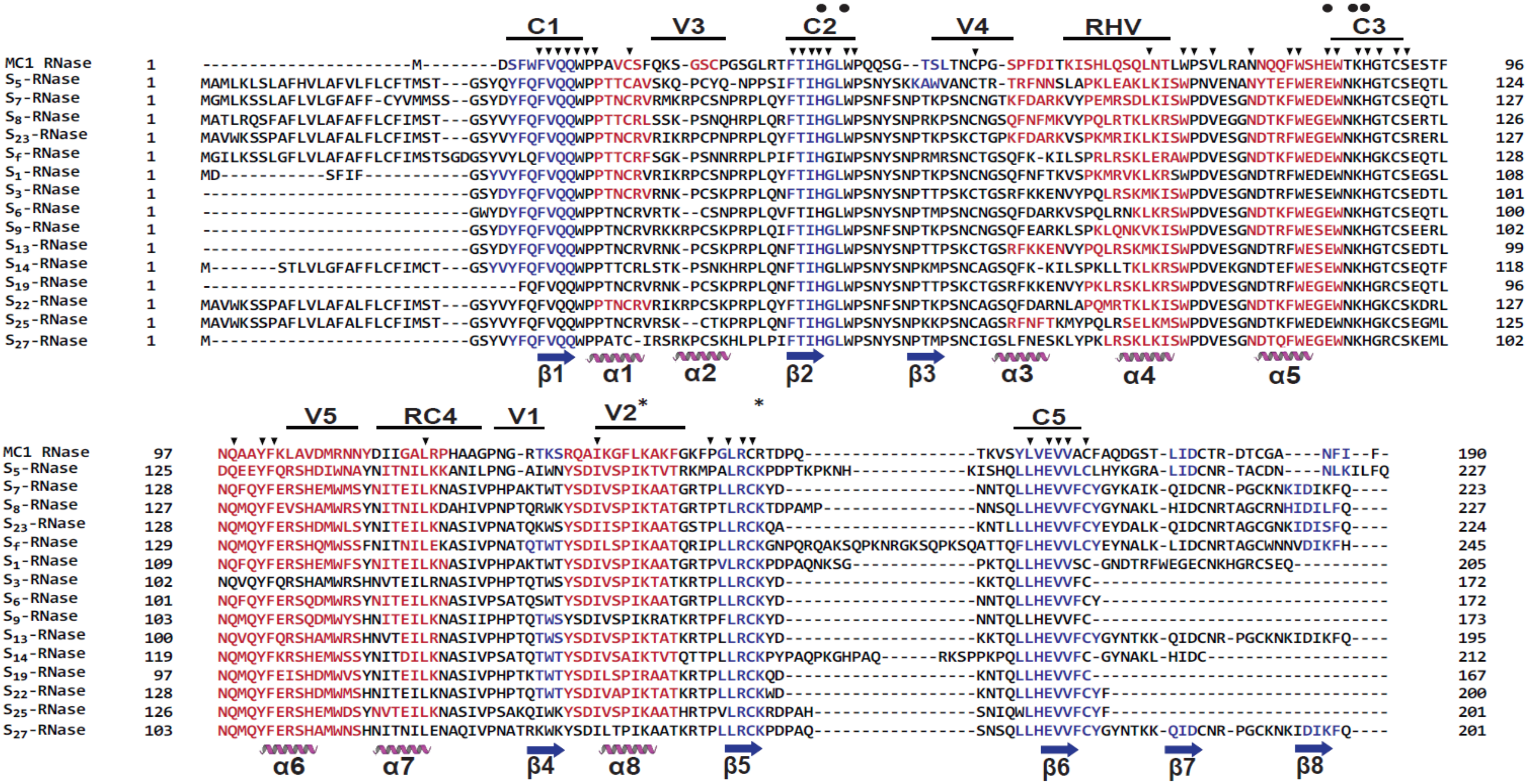

Alignment of 15 almond S-RNase protein sequences with the sequence of a template bitter gourd seed RNase (MC1 RNase). In each S-RNase sequence, the positions of predicted secondary structure elements are indicated by arrows and blue text (β-sheets) or helices and red text (α-helices). Conserved regions (C1, C2, C3, RC4 and C5) and variable regions (V1 to V5) are labelled. Absolutely conserved residues are indicated by arrowheads above the alignment. Two active site residues in the C2 region (a histidine (H) and a tryptophan (W)) and three active site residues in the C3 region (a glutamic acid (E), a lysine (K) and a histidine (H)) that were identified in relation to other *Prunus S* alleles (S_3-RNase of *Pyrus pyrifolia*) are indicated by black circles. Conserved lysine (K) residues that were identified corresponding to the other gametophytic self-incompatible species (S_3L-RNase of *Petunia inflata*) are indicated by asterisks.

Figure S12

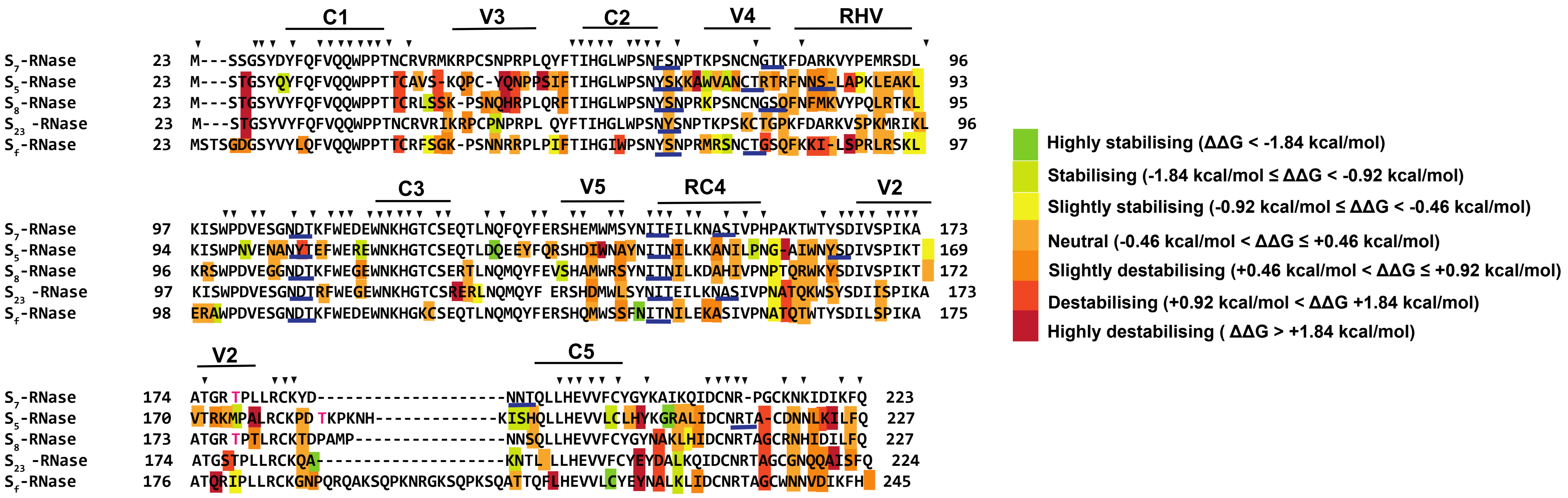

Alignment of five almond S-RNase sequences, showing the predicted effects on protein stability for specific mutations. Conserved regions (C1, C2, C3, RC4 and C5) and variable regions (V1 to V5) are labelled. Absolutely conserved residues are indicated by arrowheads above the alignment. The predicted effects of replacing specific residues in the S_7-RNase by the residues present at the corresponding position in other S-RNases are shown using a colour scale from green (highly stabilising) to red (highly destabilising). Predicted *N*-glycosylation sites (sequons) are underlined in blue and *O*-glycosylation sites are shown in pink.

Figure S13

```
>S1
TTATGTCTATTTACAATTTGTGCAACAATGGCCACCGACAAACTGCAGAGTTCGCATCAAGCGACCTTGCTCCAATCCCCGGCCACTACAATATTTCACC
ATCCATGGCCTATGGCCAAGTAACTATTCAAACCCAACGAAGCCCAGTAATTGCAATGGGTCGCAATTTAACTTTACGAAAGTGGTATGATTATTTCAAA
TTTTTTTTT

>S3
TTATGACTATTTTCAATTTGTGCAACAATGGCCACCGACCAACTGCAGAGTTCGAACGAAATGCTCCAAGCCCCGGCCATTACAATATTTCACCATCCAT
GGCCTATGGCCAAGTAATTATTCAAACCCAACGATGCCCAGTAATTGCAATGGGTCGAAATTTGATGACAGGAACGTGGTATGTATTGTTTCATTATTTT
TTCACTTACTCTTTAGC

>S5
ATAATTTTGGCAGGATCTTATGACTATTTTCAATTTGTGCAACAATGGCCACCGACCAACTGCACAGTTAGCAAACAACCTTGCTACCAAAACCCGCCCT
CAATTTTCACCATCCATGGCCTATGGCCAAGTAATTATTCCAAAAAGGCTTGGGTGGCGAATTGCACTCGGACGCGATTTAACAATAGTTTGGTATGAAT
TGGCTCTTTGTTTTTCTAG

>S7
CACAATAATTTTGGCAGGATCTTATGACTATTTTCAATTTGTGCAACAATGGCCACCGACCAACTGCAGAGTTCGCATGAAGCGACCTTGCTCCAATCCC
CGGCCATTACAATATTTCACAATCCATGGCCTATGGCCAAGTAATTTTTCAAACCCAACGAAGCCCAGTAATTGCAATGGGACTAAATTTGATGCAAGGA
AAGTGGTACGTATTGATTT

>S8
TCACAATAATTTTCGCAGGATCTTACGTGTATTTTCAATTTGTGCAACAATGGCCACCGACGACCTGCAGACTTAGCAGCAAACCTTCCAACCAACACCG
GCCATTACAAAGATTCACCATCCATGGCCTATGGCCAAGTAACTATTCAAACCCAAGGAAGCCCAGTAATTGCAATGGGTCACAATTTAACTTTATGAAA
GTGGTATGTATTGTTTCTTTTCTTTT

>S9
CAATTTGTGCAACAATGGCCACCGACTAACTGCAGAGTTCGCAATAAACCTTGCTCCAAACCCCGGCCATTACAAATCTTCACCATCCATGGCCTATGGC
CAAGTAATTATTCAAACCCAACGAAGCCCAGTAATTGCAATGGATCCCAATATGAGGCAAGGAAATTGGTATGTATTGTTTGTGTTCTTTTTTGACTTAT
TCTTTAGTGTCTAGTTTTT

>S23
AATAATTTTTGCAGGATCTTATGTCTACTTTCAATTTGTGCAACAATGGCCACCGACCAACTGCAGAGTTCGCATCAAACGACCTTGCCCCAATCCCCGA
CCATTACAATATTTCACCATCCATGGCCTATGGCCAAGTAATTATTCAAACCCAACGAAGCCCAGTAAATGCACTGGTCCGAAATTTGATGCAAGGAAAG
TGGTATGTATTGCTTCTTTTT

>S25
AAAAAATTTGGCAGGGTCTTATGACTATTTTCAATTTGTGCAACAATGGCCACCGACCAACTGCAGAGTTCGCTCGAAATGCACCAAACCCAGGCCGTTA
CAAAACTTCACCATCCATGGCCTATGGCCAAGTAATTATTCAAACCCAAAGAAGCCCAGTAATTGCGCTGGGTCGCGATTTAACTTTACTAAAATGGTAT
GCACTGTTTCGTATTTTTTAAATACTT

>Sf
TTTCGCAGGATCTTATGTCTATTTACAATTTGTGCAACAATGGCCACCGACCACCTGCAGATTTAGCGGCAAACCTTCCAACAACCGCCGTCCATTACCA
ATTTTCACCATCCATGGCATATGGCCAAGTAATTATTCAAACCCAAGGATGCGCAGTAATTGCACTGGGTCGCAATTTAAAAAAATATTGGTATGTATTG
TGTTTGTTTTTTTTTAAC
```

DNA sequences synthesised for evaluation of primer sets WriPdSf-2, WriPdSf-3, WriPdSf-4 and WriPdSf-5, with primer annealing sites underlined
